# The Fraction of Cancer Attributable to Ways of Life, Infections, Occupation, and Environmental Agents in Brazil in 2020

**DOI:** 10.1371/journal.pone.0148761

**Published:** 2016-02-10

**Authors:** Gulnar Azevedo e Silva, Lenildo de Moura, Maria Paula Curado, Fabio da Silva Gomes, Ubirani Otero, Leandro Fórnias Machado de Rezende, Regina Paiva Daumas, Raphael Mendonça Guimarães, Karina Cardoso Meira, Iuri da Costa Leite, Joaquim Gonçalves Valente, Ronaldo Ismério Moreira, Rosalina Koifman, Deborah Carvalho Malta, Marcia Sarpa de Campos Mello, Thiago Wagnos Guimarães Guedes, Paolo Boffetta

**Affiliations:** 1 Instituto de Medicina Social, University of Rio de Janeiro State, Rio de Janeiro, Rio de Janeiro State, Brazil; 2 Pan-Americana Health Organization, Brasília, Distrito Federal, Brazil; 3 AC Camargo Cancer Center, Hospital AC Camargo, São Paulo, São Paulo State, Brazil; 4 International Prevention Research Institute, Lyon, France; 5 National Cancer Institute, Ministry of Health, Rio de Janeiro, Rio de Janeiro State, Brazil; 6 Department of Preventive Medicine, School of Medicine, University of São Paulo, São Paulo, São Paulo State, Brazil; 7 National School of Public Health, Oswaldo Cruz Foundation, Ministry of Health, Rio de Janeiro, Rio de Janeiro State, Brazil; 8 Joaquim Venâncio Polytechnic School of Health, Oswaldo Cruz Foundation, Ministry of Health, Rio de Janeiro, Rio de Janeiro State, Brazil; 9 Nursing School, Federal University of Rio Grande do Norte, Natal, Rio Grande do Norte, Brazil; 10 National Institute of Infectolgy, Oswaldo Cruz Foundation, Ministry of Health, Rio de Janeiro, Rio de Janeiro State, Brazil; 11 Health Surveillance Secretariat, Ministry of Health, Brasília, Federal District, Brazil; 12 The Tisch Cancer Institute, Icahn School of Medicine at Mount Sinai, New York, New York, United States of America; Indian Institute of Toxicology Research, INDIA

## Abstract

Many human cancers develop as a result of exposure to risk factors related to the environment and ways of life. The aim of this study was to estimate attributable fractions of 25 types of cancers resulting from exposure to modifiable risk factors in Brazil. The prevalence of exposure to selected risk factors among adults was obtained from population-based surveys conducted from 2000 to 2008. Risk estimates were based on data drawn from meta-analyses or large, high quality studies. Population-attributable fractions (PAF) for a combination of risk factors, as well as the number of preventable deaths and cancer cases, were calculated for 2020. The known preventable risk factors studied will account for 34% of cancer cases among men and 35% among women in 2020, and for 46% and 39% deaths, respectively. The highest attributable fractions were estimated for tobacco smoking, infections, low consumption of fruits and vegetables, excess weight, reproductive factors, and physical inactivity. This is the first study to systematically estimate the fraction of cancer attributable to potentially modifiable risk factors in Brazil. Strategies for primary prevention of tobacco smoking and control of infection and the promotion of a healthy diet and physical activity should be the main priorities in policies for cancer prevention in the country.

## Introduction

It has been recognized for several decades that many human cancers are caused by external factors and therefore can—at least in theory—be prevented [1]. A large number of modifiable factors, including environmental agents, microbes, and personal behaviors, have been classified as established human carcinogens by the International Agency for Research on Cancer (IARC), and more are suspected carcinogens [2]. In parallel, understanding of carcinogenesis has progressed, and despite remaining uncertainties, this has helped to base cancer prevention on reliable quantitative data [3]. Therefore, a significant number of cases could be prevented by avoiding or reducing exposure to many known risk factors. Despite a growing body of scientific evidence on the causes of cancer, in many countries there are no quantitative estimates of the potential for cancer prevention associated with these risk factors.

In 1981, Doll and Peto [4] estimated that 30% of cancer deaths in the of estimated cancers in male United States (US) were linked to tobacco smoking. In Nordic countries, 33% adults and 20% in female adults were due to exposure to active and passive tobacco smoking, alcohol use, asbestos and other occupational carcinogens, ionizing and solar radiation, obesity, human papillomavirus and *Helicobacter pylori* infection [3]. In France, in 2000, 35% of cancer deaths were attributable to known risk factors including active and passive tobacco smoking, alcohol use, infections, excess weight, physical inactivity, reproductive history, use of exogenous hormones, and exposure to ultraviolet radiation, occupational agents and asbestos [5,6]. In the United Kingdom (UK), in 2010, 14 risk factors (tobacco smoking, alcohol use, diet, and excess weight, among others) accounted for 42.7% of cancer cases [7]. In Japan, 55% of cancers in male adults were estimated as preventable [8].

Rapid urbanization in low- and middle-income countries has increased the number of individuals exposed to cancer risk factors. In China, an analysis of causes of cancer pointed out that 60% of cancer deaths were due to modifiable risk factors [9]. In South Korea, 25% of cancer cases and deaths were estimated as preventable by means of actions to reduce exposure to infectious agents [10]. Danaei et al. [11] showed that mortality from 12 types of cancer were attributable to nine behavioral and environmental risk factors, and preventative measures would prevent 35% of cancer deaths worldwide, with 34% in low-and middle-income countries and 37% in high-income countries.

Brazil is characterized by an environment combining poor living conditions in low-income areas with the effects of rapid demographic and nutritional transition, which increases the population’s exposure to cancer risk factors [12]. Death rates from breast, colorectal, prostate and female lung cancer have increased, while gastric, cervical, and male lung cancer have declined, although they still remain high [13].

Some Brazilian studies have reported high proportions of mortality and incidence of cancer attributable to tobacco smoking [14–17], particularly for lung and larynx cancers.

The aim of this study was to estimate attributable fractions of cancers in the adult population resulting from exposure to tobacco smoking, alcohol use, diet, overweight and obesity, physical inactivity, infections, reproductive history, and selected occupational and environmental agents.

## Materials and Methods

### Selected risk factors and types of cancer included in the study

Only risk factors known as potentially preventable (i.e., modifiable) causes associated with cancer were selected for this study. The selection of risk factors was also based on the availability of information required to estimate the level of exposure in the Brazilian population. The study included human carcinogens listed in Group 1 of the International Agency for Research on Cancer (IARC) [2], as well as factors showing evidence of probable causes (e.g., diet, nutrition) as reported by the World Cancer Research Fund/American Institute for Cancer Research [18], and potential cancer preventive factors evaluated in the series of the IARC Handbooks of Cancer Prevention [19]. Other risk and protective factors, such as physical inactivity and air pollution, were identified and selected from the most recent high quality meta-analyses when they showed significant relative risks [20–24].

Risk factors selected for this study were associated with 25 anatomical cancer sites that were considered for the analysis.

All risk factors with their respective optimum exposure levels and associated cancers included in the study are presented in Table 1.

**Table 1:** Risk factors, theoretical optimum exposure level, and associated cancers.

Exposure	Optimum exposure level	Associated cancer
**Tobacco smoking (active)**	Never smoking	Oral cavity, Esophageal (squamous and adenocarcinoma), Stomach (cardia and non-cardia), Liver, Pancreas, Larynx, Lung, Ovary, Kidney, Bladder
**Frequent alcohol consumption**	No alcohol consumption	Oral cavity, Esophageal (squamous) Stomach (non-cardia), Colon and rectum, Liver, Larynx, Breast (postmenopausal)
**Diet**		
***Low intake of fruits and vegetables***	>160 g of fruits/day; >240 g of vegetables/day	Oral cavity, Esophageal (squamous), Stomach (cardia and Non-cardia portions), Larynx, Lung
***Intake of processed meat***	No consumption	Stomach (Cardia and non-cardia portions), Colon and rectum
***Excess intake of red meat***	Up to 70 g/day	Colon and rectum
***Excess intake of salt***	Up to 10 g/day	Stomach (cardia and con-cardia portions)
**Overweight/obesity**	BMI < 25 kg/m^2^	Colon and rectum, Esophageal (adenocarcinoma), Gallbladder and bile ducts, Breast (postmenopausal), Corpus uterus, Kidney
**Physical inactivity**	Practice of physical activity at leisure-time and/or domestic-related and/or work-related and/or commuting-related	Colon and rectum, Pancreas, Breast, Corpus uterus, Prostate, Kidney
**Infectious agents**	No infection	
***Human papillomavirus (HPV)***		Oral cavity, Oropharynx, Larynx, Cervix uterus, Penis
***Helicobacter pylori (H*. *pylori)***		Stomach (non-cardia portion)
***Epstein-Barr virus (EBV)***		Nasopharynx, Hodgkin lymphoma, Non-Hodgkin lymphoma
***Hepatitis B virus (HBsAg)***		Liver
***Hepatitis C antibody (anti-HCV)***		Liver
**No or short breastfeeding**	Breastfeeding for more than 6 months	Breast
**Use of oral contraceptives**	No use	Breast
**Occupational agents** (Formaldehyde, Painting, Rubber industry, Benzene, Leather dust, Silica, Wood dust, Nickel, Asbestos, Benzopyrene, Diesel, Iron/steel, Radon, Gamma radiation)	No exposure	Esophageal (squamous), Nasopharynx, Sinonasal, Larynx, Lung, Bladder, Breast, Ovary, Mesothelioma, Non-Hodgkin lymphoma, Leukemia
**Environmental agents**		
***Solar radiation*** (frequent exposure and/or sunburn episodes among white-skinned population)	No daily exposure or history of sunburn	Melanoma
***Second-hand smoke***	No exposure	Lung
Particulate matter (PM10) pollution*	≤ 20 μg/m^3^	Lung

*Among population living in urban areas.

### Prevalence of exposure to the studied risk factors

The estimates of prevalence of exposure to lifestyle and reproductive factors in individuals aged 30 years and over were taken from the most important and nationwide population-based surveys carried out in Brazil by the Ministry of Health and by the Brazilian Institute of Geography and Statistics (IBGE) between 2006 and 2008 [25]. The following surveys were considered: National Household Sample Survey (PNAD), 2008 [26]; Family Budget Survey (POF), 2008–2009 [27]; Special Survey on Tobacco Use in Brazil (PETab), 2008 [28]; Telephone-based Surveillance of Risk and Protective Factors for Chronic Diseases (VIGITEL), 2008 [29] and National Demographic and Health Survey of Children and Women (PNDS), 2006 [30]. More details about these surveys, including the access to questionnaires, can be seen in S1 and S2 Tables.

The prevalence of the exposure of adults to hepatitis viruses was taken from a population-based survey conducted in all Brazilian capitals in 2005 (Population-based Study of Hepatitis A, B and C Infection Prevalence in Brazilian Capital Cities) [31]. The prevalence of the other infectious agents were obtained from Brazilian epidemiological studies on Human papillomavirus HPV [32,33], Helicobacter pylori (H. pylori) [34], and Epstein-Barr virus (EBV) [35].

The definitions of dietary intake exposures were taken from the recommendations of the World Health Organization (WHO) and Food and Agriculture Organization for the United Nations (FAO) [36]. The maximum consumption for red meat was considered as 70 g/day, and a minimum consumption of 160 g/day and 240 g/day was considered for fruits and vegetables, respectively.

Estimates of the prevalence of workers with occupational exposures were obtained from data available in the 2000 Brazilian Census [37] and in the 2003 National Household Sample Survey (PNAD, 2003) [38]. The occupations for each field based on IBGE’s occupational classification with exposure to carcinogenic agents according to IARC (Group 1) [2], as listed in Table 1, were included in the study.

Sources of environmental exposure data included surveys and studies supporting approximate estimates of background population exposures.

The population exposed to particulate matter (PM10) pollution was estimated as being the proportion of individuals living in metropolitan areas in Brazil in 2008 [39]. The average annual exposure to PM10 in these areas was considered similar to the weighted average exposure in 21 industrialized cities obtained by measurements of the Brazilian Environment Protection Agency’s pollution monitoring programs between 2000 and 2008 [39]. The estimated exposure to PM10 was then calculated considering the percentage of the population living in all metropolitan areas of the country, and the risk was accounted for when the exposure was higher than the maximum annual average exposure of 20 μg/m^3^, according to WHO recommendations [40].

Exposure to passive tobacco smoking was calculated as the proportion of individuals aged 30 years and over who reported being exposed to second-hand tobacco smoke indoors in the PETab [28].

Exposure to solar radiation was based on the proportion of white-skinned individuals in 2008 [26]. The prevalence of individuals exposed to high levels of sun exposure and with a history of sunburn was considered among the white-skinned population. The number of individuals with high levels of sun exposure was drawn from a population-based survey conducted in Brazilian cities between 2003 and 2005 [41] and the history of episodes of sunburn was obtained from a Brazilian study conducted in the state of Rio Grande do Sul, which has a large number of white-skinned individuals [42]. These proportions were multiplied by the percentage of white-skinned individuals living in Brazil in 2008 [26].

All estimates of prevalence or average consumption defined for the study with their respective data sources are presented in S1 and S2 Tables.

### Selection of risk estimates

The risk estimates for the selected risk factors applied in the calculation of population-attributable fractions (PAF) were obtained from literature reviews. Separate reviews of studies were conducted for each group of risk factors, especially taking into account meta-analyses studies. When there were no meta-analyses available, the selection of relative risks was based on international and Brazilian observational studies with high quality standards and involving a sufficient number of participants to allow inference on risk associations. All risk estimators and respective references can be found in S1 and S2 Tables.

### Statistical analysis

The PAF for categorical variables were estimated using the following equation [43]:

$$PAF=\frac{P_{e}\left(RR-1\right)}{P_{e}\left(RR-1\right)+1}$$

Where P_e_ = prevalence of the exposure in the population and RR = relative risk for the cancer associated with the exposure

For RR with continuous exposures such as fruits, vegetables, processed meat, red meat, and PM10, the following equation was applied:

$$PAF=\frac{\text{exp}\left[In\left(RRunit\right)\;\times \;\bar{x}\right]\;-1}{\text{exp}\left[In\left(RRunit\right)\;\times \;\bar{x}\right]}$$

Where *RR*_unit_ = relative risk for each unit increment in the exposure and $\bar{x}=\text{ average exposure}$

The joint effect in the presence of more than one biologically independent risk factor was estimated by the equation proposed by Ezzati et al. [44]:

$$PAF=1-\Pi_{i=1}^{n}\left(1-PAF_{i}\right)$$

Where: *PAF*_*i*_ = individual risk factors PAF.

The prevalence of exposures was selected for the 2000–2008 period and the number of deaths and new cases of cancer for the population aged 30 years and over was estimated for the year 2020, in order to keep a 12–20 year lag-time period between exposures and outcomes.

The projection of cases was based on data from the Brazilian Population-Based Cancer Registries (PBCR) [45] and from the Brazilian Mortality Information System [46]. Mortality data for the 1996–2011 period were corrected for underreporting of deaths. Given that the demographic methods for estimating the level of mortality of the adult population yielded inconsistent results, correction factors for the underreporting of deaths according to year, sex, age group, and States of the Federation were estimated in order to obtain life expectancy by year, sex, and state provided by the Brazilian Bureau of statistics. A set of nonspecific cancers together with ill-defined causes were distributed proportionately, according to the state of residence, year, sex, age, and causes of death. After applying a three-year moving average on the population and a mortality time series, rates were calculated and projected for each type of cancer using the geometric growth rate for the period. The mortality for 2020 was projected using a linear regression model with its naperian logarithm as the response variable. Lastly, the incidence for each type of cancer was obtained by multiplying mortality by the average incidence: mortality (I/M) ratio estimated from 11 pooled PBCR with the best data quality in the period from 2000 to 2010. The criteria to select these PBCR were: population size greater than 200,000; reporting data from at least three consecutive years; information with death-certificate-only registrations less than 20%; more than 70% morphologically-verified cases (MV > 70%) and no unexpected fluctuations in the number of incident cases (stable coverage).

This study was approved by the Research and Ethics Committee of the Institute of Social Medicine of the Rio de Janeiro State University in 20 August 2013 (Process Number: CAAE 18415713.2.0000.5260).

## Results

**Fig 1:**
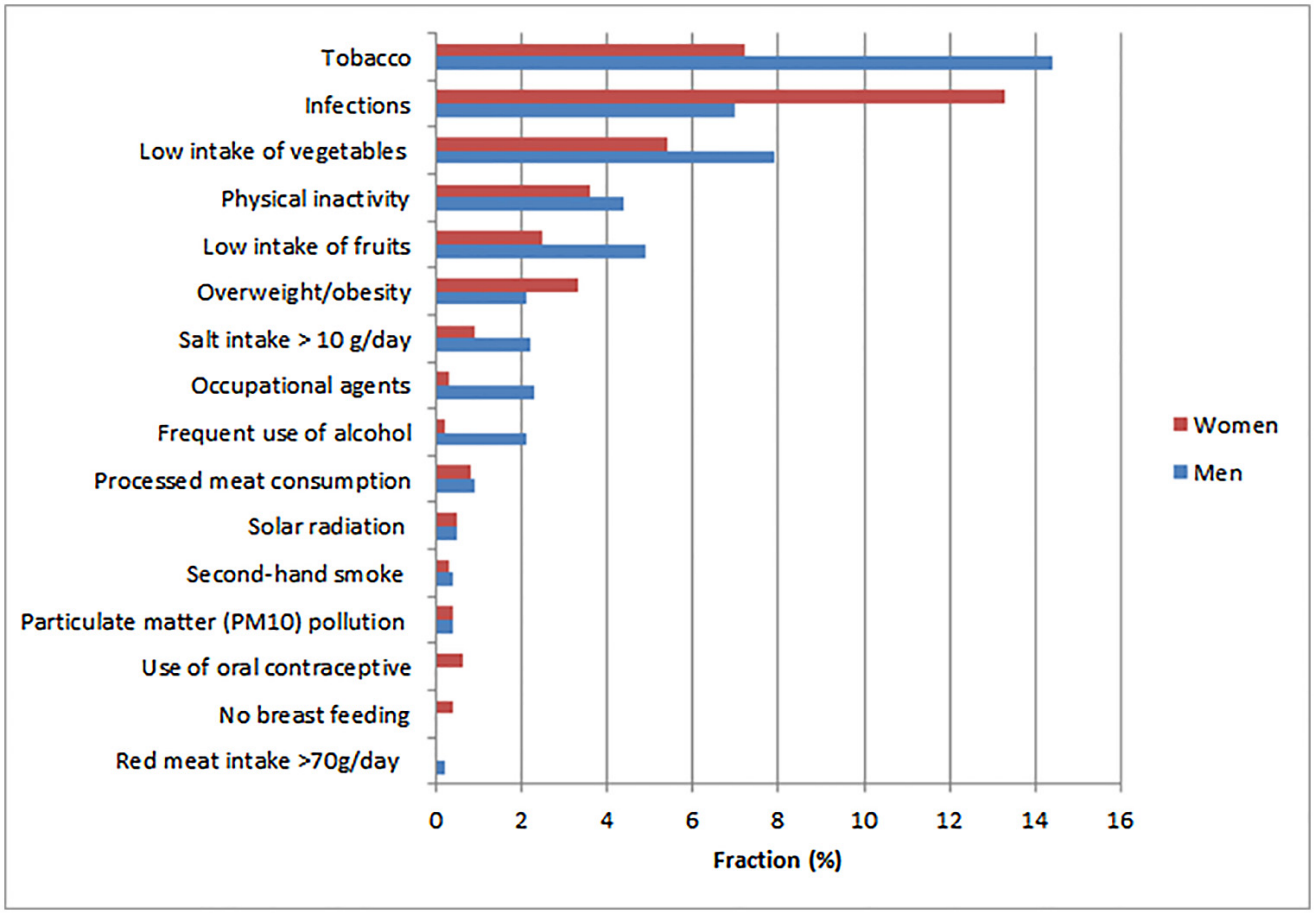
Estimated fraction of the total cases of cancer for the population 30 years old and over in Brazil attributable to selected risk factors in 2020.

The fraction of cancers attributable to each risk factor in relation to the total number of cases by sex can be seen in Fig 1.

The highest fraction is due to tobacco smoking, infections, low consumption of fruits and vegetables, physical inactivity and overweight/obesity.

The combined effect of the studied risk factors for each type of cancer by sex are shown in Table 2. High proportions of preventable deaths and cases were estimated for cervical, breast, lung, colon, and rectal cancers. Details of the estimated specific and combined PAF by cancer site are described in S1 and S2 Tables.

**Table 2:** Population-attributable fractions (PAF) and corresponding estimates for 2020 of deaths and cases by cancer site for the population 30 years old and over in Brazil by sex.

		Men			Women	
Cancer site	PAF (%)	Deaths	Cases	PAF (%)	Deaths	Cases
Cervix uteri				100.0	8718	23773
Oral cavity	95.0	3877	10624	92.3	1522	5954
Nasopharynx	89.2	295	651	89.2	159	387
Trachea, bronchus and lung	88.3	17206	20071	73.6	11680	14581
Stomach (non-cardia portion)	88.0	7645	11410	83.6	3927	6396
Larynx	80.2	4104	7035	71.1	531	1321
Esophagus (squamous)	72.8	4915	6783	60.2	1178	1868
Non-Hodgkin lymphoma	71.5	2004	4747	71.0	1670	4039
Stomach (cardia portion)	65.5	1950	2905	62.2	1013	1650
Hodgkin lymphoma	61.8	135	507	61.8	108	455
Corpus uteri				44.8	960	3548
Esophagus (adenocarcinoma)	44.2	753	1039	43.6	215	341
Colon and rectum	43.8	6121	13760	25.3	3570	7808
Breast (post menopause)				17,3	2475	10349
Pancreas	33.9	1973	1973	24.7	1632	1632
Mesothelioma	32.7	31	37	5.6	3	5
Kidney	35.6	997	2500	31.3	466	1253
Melanoma of skin	27.2	342	1371	26.2	238	1300
Breast (pre menopause)				11.2	538	2251
Liver	25.9	1389	1389	23.9	802	802
Bladder	23.6	815	2558	17.7	272	833
Nose sinuses	21.7	47	163	6.4	10	43
Leukemia	16.8	598	779	3.9	126	161
Oropharynx	14.4	658	1034	14.4	142	275
Penis	9,6	51	231			
Gallbladder and bile ducts	6.6	89	102	18.2	432	481
Prostate	6.0	1363	6801			
Ovary				1.5	75	142
All above		57808	98890		42676	92067
All sites*		128626	292156		110744	265452
***Total reduction (%)***		**44.9**	**33.9**		**38.5**	**34.7**

*Excluding non-melanoma skin cancer.

## Discussion

This is the first study to estimate the fraction of cancers attributable to a wide range of potentially modifiable risk factors in Brazil. The selected risk factors may explain 34.2% of cancer cases and 42.0% of cancer deaths across the country in 2020. These fractions are lower than those reported in China for deaths (58%) [9], in the US for incident cases (60%) [47], in Japan for deaths and cases (46.2% and 42.7%, respectively) [8], and for incident cases in the UK (42.7%) [7]. Our values are similar to those estimated for cases in Australia (32.0%) [48], but higher than those estimated for deaths in France (35.0%) [6] and for cases in Nordic countries (26.5%) [5] (Table 3). Although all these studies evaluated the fraction of cancers attributable to potentially preventable risk factors, they did not include exactly the same risk factors and for that reason, this comparison should be regarded with caution.

**Table 3:** Comparison of estimates of cancer deaths and incident cases attributable to the combined effects of modifiable risk factors among different countries.

Country	PAF* (%)	
	Deaths	Cases
Brazil, 2020 (present study)	42.0	34.2
France, 2000^a^ [6]	35.0	
Nordic countries, 2000^a^^,^^b^ [5]		26.5
Japan, 2005^c^ [8]	46.2	42.7
China, 2005 [9]	57.4	
United States of America, 2008 [47]		60.0
United Kingdom, 2010 [7]		42.7
Australia, 2010^c^ [48]		32.0

*Population attributable fraction.

The estimates did not include:

^a^diet risk factors;

^b^physical inactivity;

^c^occupational agents.

Tobacco smoking is the leading contributor to the incidence of cancer and deaths expected for 2020 in Brazil. The fraction attributable to infection appears as the second preventable cause of cancer as observed in low- and middle-income countries [49]. The high number of preventable cases of cancer due to infections among women is affected by the incidence of cervical cancer, which is particularly high in the less developed regions of the country [13].

The percentage of adult individuals with positive tests for HBsAg (0.6%) and for anti-HCV (1.6%) used in our study can be considered low if compared to other countries [46]. Even if a potential bias of under-detection could not be excluded, we still opted to use the data from the hepatitis survey in Brazilian capitals [31], given that it is clearly more representative than other epidemiological studies restricted to small areas and that its results were not different from what was detected among blood donors in the State of São Paulo [50].

Because of the urbanization and industrialization progress in Brazil, poor diet and excess weight are among the main risk factors leading to new cases of cancer. This scenario is highly concerning because the food industry in low- and middle-income countries like Brazil is pushing hard to increase the demand for food products [51] which are high in fat, sugar, calories, and salt, and also include carcinogenic compounds such as nitrites and nitrates. At the same time, the consumption of fruits and vegetables that may protect against several cancers is very low in Brazil [52], below one-third of the minimum recommended level of 400 g/day [36]. Furthermore, the increasing intake of high-energy dense food products and sugar-added beverages has contributed to the increase in the prevalence of overweight/obesity [27], accounting for a significant fraction of cancer cases in Brazil. Also important is the high intake of salt in the Brazilian population leading to an expressive PAF for stomach cancer. Although mortality trends for stomach cancer have been decreasing in all regions of the country, death rates still rank third among men and fifth among women [13].

Estimates of PAF for overweight and obesity were higher than those reported in other countries. In Japan, the country with the lowest average body mass index (BMI) in the high-income world, excess weight accounted for about 1% of cancer cases [8]. In France, the country with the fourth lowest average BMI among high-income countries [49], the proportion of cancer attributable to excess weight was 1.1% (1.6 among males and 2.3% among female adults) [6]. Brazil is undergoing a displacement of healthy foods and eating practices, leading to a rapid increase in overweight and obesity [27]. Recent data show that about one-third of children, one-fifth of adolescents, and half of adults have excess weight in Brazil [27]. Unless effective measures are put in place to control and reduce the dramatically increasing prevalence of overweight and obesity, a much greater number of obesity-related cancers may be expected.

Our estimates for physical inactivity (4.0%), lower than what was reported in Canada (7.9%) [53], and higher than those seen in Korea [54] and France [6], are consistent with the recent literature [55] and may play a role in the burden of cancer in Brazil related to physical inactivity, especially for breast and colon cancer.

The ongoing demographic transition in Brazil has led to important lifestyle changes, especially among female adults. The increase in life expectancy with a markedly reduced fertility is contributing to a higher level of exposure to reproductive risk factors for breast cancer [56]. As many of these reproductive factors are not subject to preventive intervention, only breast-feeding and the use of oral contraceptives were included in the present study. Although the use of oral contraceptives and not breast-feeding are prevalent conditions among Brazilian women, small attributable fractions were estimated (2.1% and 1.4%, respectively, as shown in S2 Table), due to the small relative risks related to these factors.

The occupational agents analyzed in the present study, despite high relative risks for some conditions, show a lower impact on total cancer in the Brazilian population (2.3% in men and 0.3% in women) when compared to data from other countries where a range from 3 to 14% in male adults and 1 to 2% in female adults was observed [57].

The low impact of the occupational agents studied is noteworthy, possibly suggesting an underestimation of the real prevalence. This fact may be explained by the use of a specific criterion for determining occupations highly exposed to agents that are classified as definitively carcinogenic. The number of workers included in this criterion was estimated based on data drawn from official statistics. Since many individuals in occupations with a high potential for carcinogenic-agent exposure work in the informal market, underreporting is likely to occur [58]. This fact may have affected, in particular, the data on workers’ exposure to asbestos.

The relative risk estimates used in this study were drawn from meta-analyses and large international studies. Although this option helps external comparison, some of these estimates may not accurately represent the exposure effects in our population. Another limitation refers to the suboptimal quality of mortality and incidence data. The techniques used to correct for underreporting and ill-defined codes of death are not sufficient to prevent underestimate of rare cancers, which depend on complex diagnostic workups. This may explain the low incidence and mortality estimates for malignant pleural mesothelioma, a neoplasm that usually depends on surgical tissue biopsies and the use of immunohistochemistry for definitive diagnosis [59]. It is also possible that the PAF to melanoma associated with sun exposure may underestimate the number of cases of the disease. This is because only the white-skinned population was considered, and because the history of sunburns was observed only in the south and may therefore not accurately represent the whole country.

The low coverage of cancer registries prevented direct incidence estimates. We also were unable to estimate time trends in incidence:mortality (I/M) ratios, as few registries have long enough series. Instead, the available data allowed us to estimate with reasonable reliability the average recent I/M ratios for the most frequent cancers, which were used in the projection of cases. Hence, the recent trends in incidence, which are not yet reflected in mortality, were not considered in our projections.

In our study, we opted to include the population aged 30 years and older considering that the cases of cancer arising before this age are more likely to be due to genetic or other unknown origins, and that they would not reflect the exposure to the studied modifiable risk factors. Additionally, we assumed that in a population aged 30 years and over, the majority of individuals would better cover potential exposures accumulated for at least 10 years during adult life. Accordingly, it must be stressed that although the estimated fractions refer to the population aged 30 years and over, the selected prevalence of the studied risk factors was based on related exposure during the full life course, not only exposure after age 30.

Despite these limitations, our data suggest various patterns that are consistent with a country in economic transition having characteristics of both developed and less developed countries. Facing poverty and marked health inequalities, low-income countries show a high prevalence of non-cardiac gastric and cervical cancers, particularly in poor areas [60], which can be prevented through actions primarily focused on tobacco control, control of infections, and healthy eating. On the other hand, cancers like breast, prostate and colorectal, common in high-income countries, demand actions to change sedentary life.

The lessons drawn from the tobacco control experience in Brazil [12] should be followed with the implementation of preventive actions focusing on other modifiable risk factors. The promotion of healthy eating, physical activity, and protection against harmful occupational and environmental exposures are necessary. Special attention must also be given to the control of infections and sanitary conditions.

Advances in some actions designed in Brazil, such as smoke-free environments and advertisement regulations [61] are in course; however, there are many other policies yet to be implemented with legislation and economic measures to promote large-scale changes with greater impact over a broader range of modifiable risk factors.

Effective prevention strategies must be based on the results of epidemiological studies allowing future comparisons and improvement of health surveillance. Priority should be given to research projects to explore cancer risks among low-income population groups who are at an increased risk and live under conditions that make them extremely vulnerable.

## Supporting Information

S1 Table Risk factors, relative risks, prevalence of exposures and specific and combined population attributable fractions by cancer sites among men. (DOC)

S2 Table Risk factors, relative risks, prevalence of exposures and specific and combined population attributable fractions by cancer sites among women. (DOC)

## References

[1] Peto J. Cancer epidemiology in the last century and the next decade. Nature. 2001;411:390–395. 11357148 10.1038/35077256

[2] International Agency for Research on Cancer. IARC Monographs on the Evaluation of Carcinogenic Risks to Humans. Vol. 1–110 Lyon, IARC, 1971–2015 Available: http://iarc.fr/en/publications/list/monographs/index.php PMC7681469 1683674

[3] Hanahan D, Weinberg RA. The hallmarks of cancer. Cell 2000;100:57–70. 10647931 10.1016/s0092-8674(00)81683-9

[4] Doll R, Peto R. The causes of cancer: quantitative estimates of avoidable risks of cancer in the United States today. J Natl Cancer Inst. 1981;66:1191–1308. 7017215

[5] Olsen JH, Andersen A, Dreyer L, Pukkala E, Tryggvadottir L, Gerhardsson de Verdier M, et al Summary of avoidable cancers in the Nordic countries. APMIS Suppl. 1997;76:141–146. 9462826 10.1111/j.1600-0463.1997.tb05617.x

[6] Boffetta P, Tubiana M, Hill C, Boniol M, Aurengo A, Masse R, et al The causes of cancer in France. Ann Oncol. 2009;20:550–555. 10.1093/annonc/mdn597 18765462

[7] Parkin DM, Boyd L, Walker LC. The fraction of cancer attributable to lifestyle and environmental factors in the UK in 2010. Br J Cancer. 2011;105:S77–81. 10.1038/bjc.2011.489 22158327 PMC3252065

[8] Inoue M, Sawada N, Matsuda T, Iwasaki M, Sasazuki S, Shimazu T, et al Attributable causes of cancer in Japan in 2005–systematic assessment to estimate current burden of cancer attributable to known preventable risk factors in Japan. Ann Oncol. 2012;23:1362–1369. 22048150 10.1093/annonc/mdr437

[9] Wang JB, Jiang Y, Liang H, Li P, Xiao HJ, Ji J, et al Attributable causes of cancer in China. Ann. Oncol. 2012;23:2983–2989. 22689178 10.1093/annonc/mds139 PMC8890481

[10] Shin A, Park S, Shin HR, Park EH, Park SK, Oh JK, et al Population attributable fraction of infection-related cancers in Korea. Ann Oncol. 2011;22:1435–1442. 20974652 10.1093/annonc/mdq592

[11] Danaei G, Vander Hoorn S, Lopez AD, Murray CJ, Ezzati M, Comparative Risk Assessment collaborating group (Cancers). Causes of cancer in the world: comparative risk assessment of nine behavioural and environmental risk factors. Lancet 2005; 366:1784–1793. 16298215 10.1016/S0140-6736(05)67725-2

[12] Schmidt MI, Duncan BB, Azevedo e Silva G, Menezes AM, Monteiro CA, Barreto SM, et al Chronic non-communicable diseases in Brazil: burden and current challenges. Lancet. 2011;377:1949–1961. 10.1016/S0140-6736(11)60135-9 21561658

[13] Azevedo e Silva G, Gamarra CJ, Girianelli, Valente JG. Tendência da mortalidade por câncer nas capitais e interior do Brasil entre 1980 e 2006. Rev. Saude Publica. 2011;45:1009–1018. 22127651 10.1590/s0034-89102011005000076

[14] Menezes AMB, Horta BL, Oliveira ALB, Kaufmann RAC, Duquia R, Diniz A, et al Risco de câncer de pulmão, laringe e esôfago atribuível ao fumo. Rev Saude Publica, 2002;36:129–134. 12045791 10.1590/s0034-89102002000200002

[15] Corrêa PCRP, Barreto SM, Passos VM. Smoking-attributable mortality and years of potential life lost in 16 Brazilian capitals, 2003: a prevalence-based study. BMC Public Health, 2009;9:1–13. 19558658 10.1186/1471-2458-9-206 PMC2711948

[16] Oliveira AF; Valente JG, Leite IC. The disease burden attributable to smoking in the state of Rio de Janeiro, Brazil in 2000. Clinics. 2008;63:215–222. 18438576 10.1590/s1807-59322008000200010 PMC2664213

[17] Pinto M, Uga MAD. Os custos de doenças tabaco-relacionadas para o Sistema Único de Saúde. Cad Saude Publica. 2010; 26:1234–1245. 20657987 10.1590/s0102-311x2010000600016

[18] World Cancer Research Fund/American Institute for Cancer Research. Food, Nutrition, Physical Activity, and the Prevention of Cancer: A Global Perspective. Washington, DC: AICR; 2007 Available: http://www.dietandcancerreport.org/cancer_resource_center/downloads/Second_Expert_Report_full.pdf

[19] International Agency for Research on Cancer (IARC). Handbooks for Cancer Prevention, Vol. 1–13 Lyon: IARC press, 1997–2011 Available: http://www.iarc.fr/en/publications/list/handbooks/index.php

[20] Behrens G, Leitzmann MF. The association between physical activity and renal cancer: systematic review and meta-analysis. Br J Cancer. 2013;108:798–811. 10.1038/bjc.2013.37 23412105 PMC3590672

[21] O'Rorke MA, Cantwell MM, Cardwell CR, Mulholland HG, Murray LJ. Can physical activity modulate pancreatic cancer risk? a systematic review and meta-analysis. Int J Cancer. 2010;126:2957–68. 10.1002/ijc.24997 19856317

[22] Moore SC, Gierach GL, Schatzkin A, Matthews CE. Physical activity, sedentary behaviours, and the prevention of endometrial cancer. Br J Cancer. 2010;103:933–8. 10.1038/sj.bjc.6605902 20877336 PMC2965881

[23] Liu Y, Hu F, Li D, Wang F, Zhu L, Chen W, et al Does physical activity reduce the risk of prostate cancer? A systematic review and meta-analysis. Eur Urol. 2011;60:1029–44. 10.1016/j.eururo.2011.07.007 21802197

[24] Hamra GB, Guha N, Cohen A, Laden F, Raaschou-Nielsen O, Samet JM, et al Outdoor particulate matter exposure and lung cancer: a systematic review and meta-analysis. Environ Health Perspect. 2014;122:906–11. 10.1289/ehp.1408092 24911630 PMC4154221

[25] Szwarcwald CL, Malta DC, Pereira CA, Vieira MLFP, Conde WL, Souza Júnior PRB, et al National Health Survey in Brazil: design and methodology of application. Ciênc Saúde Coletiva. 2014;19:333–42. 10.1590/1413-81232014192.14072012 24863810

[26] Brasil. Instituto Brasileiro de Geografia e Estatística (IBGE). Pesquisa Nacional por Amostra de Domicílios, PNAD, 2008. [National Household Sample Survey, PNAD 2008]. Rio de Janeiro: IBGE; 2008.

[27] Brasil. Instituto Brasileiro de Geografia e Estatística (IBGE). Pesquisa de Orçamentos Familiares 2008–2009: despesas, rendimentos e condições de vida—POF, 2008–2009. [Family Budget Survey, 2008–2009]. Rio de Janeiro: IBGE; 2010.

[28] Brasil. Instituto Brasileiro de Geografia e Estagística (IBGE). Pesquisa especial de tabagismo—PETab. [Special Survey on Tobacco Use in Brazil, 2008]. Rio de Janeiro: IBGE; 2009.

[29] Brasil. Ministério da Saúde. Secretaria de Vigilância em Saúde. Vigitel Brasil 2008: Vigilância de fatores de risco e proteção para doenças crônicas por inquérito telefônico—Vigitel, 2008. [Telephone-based survey of risk factors for chronic diseases, 2008]. Brasília: Ministério da Saúde, Secretaria de Vigilância em Saúde; 2009.

[30] Brasil. Ministério da Saúde. Pesquisa Nacional de Demografia e Saúde da Criança e da Mulher—PNDS 2006: dimensões do processo reprodutivo e da saúde da criança. [National Survey of Demography and Health, 2006]. Brasília: Ministério da Saúde; 2009.

[31] Brasil. Universidade de Pernambuco. Núcleo de Pós-Graduação. Estudo de prevalência de base populacional das infecções pelos vírus das hepatites A, B e C nas capitais do Brasil. [Study of population based prevalence of hepatitis virus A, B and C infection in Brazilian capitals]. Relatório de Pesquisa Brasil; 2010.

[32] Giuliano AR, Lazcano-Ponce E, Villa LL, Flores R, Salmeron J, Lee JH, et al The human papillomavirus infection in men study: human papillomavirus prevalence and type distribution among men residing in Brazil, Mexico, and the United States. Cancer Epidemiol Biomarkers Prev. 2008;17:2036–43. 18708396 10.1158/1055-9965.EPI-08-0151 PMC3471778

[33] Girianelli VR, Thuler LCS, Azevedo e Silva G. Prevalência de HPV em mulheres assistidas pela estratégia saúde da família na Baixada Fluminense do Estado do Rio de Janeiro / Prevalence of HPV infection among women covered by the family health program in the Baixada Fluminense, Rio de Janeiro, Brazil. Rev Bras Ginecol Obstet. 2011;32:39–46. 10.1590/s0100-72032010000100007 20209261

[34] Rodrigues MN, Queiroz DMM, Rodrigues RT, Rocha AMC, Luz CRL, Braga LLBC. Prevalência da infecção pelo Helicobacter pylori em Fortaleza, Ceará. Rev Saúde Publica. 2005;39:847–849. 16254664 10.1590/s0034-89102005000500022

[35] Figueira-Silva CM Pereira FEL. Prevalence of Epstein-Barr virus antibodies in healthy children and adolescents in Vitória, state of Espírito Santo, Brazil. Rev Soc Bras Med Trop. 2004;37:409–412. 15361959 10.1590/s0037-86822004000500008

[36] World Health Organization (WHO)/ Food and Agriculture Organization for the United Nations (FAO). Expert Report on Diet, Nutrition and the Prevention of Chronic Diseases. WHO Technical Report Series 916. Geneva: WHO/FAO; 2003.

[37] Brasil. Instituto Brasileiro de Geografia e Estatística (IBGE). Censo Demográfico, 2000. Pessoas ocupadas por atividade no Brasil e grandes regiões. [Demographic Census-Brazil, 2000]. Rio de Janeiro: IBGE; 2001.

[38] Brasil. Instituto Brasileiro de Geografia e Estatística (IBGE). Pesquisa Nacional por Amostra de Domicílios, PNAD, 2003. [National Household Sample Survey, 2003]. Rio de Janeiro: IBGE; 2004.

[39] Freitas CU, Junger W, de Leon AP, Silva MAFR, Gouveia N. Poluição do ar em cidades brasileiras: selecionando indicadores de impacto na saúde para fins de vigilância. Epidemiol Serv Saude. 2013;22:445–454.

[40] World Health Organization (WHO). Recommendations on Ambient Air Quality. Pollytion Atmosphérique—numéro spécial, November 2012.

[41] Szklo AS, Almeida LM, Figueiredo V, Lozana JA, Mendonça GAS, Moura L. Comportamento relativo à exposição e proteção solar na população de 15 anos ou mais de 15 capitais brasileiras e Distrito Federal, 2002–2003. Cad Saude Publica. 2007;23:823–834. 17435880 10.1590/s0102-311x2007000400010

[42] Bakos L, Mastroeni S, Bonamigo RR, Melchi F, Pasquini P, Fortes C. A melanoma risk score in a Brazilian population. An Bras Dermatol. 2013;88:226–232. 10.1590/S0365-05962013000200007 23739694 PMC3750885

[43] Levin M. The occurrence of lung cancer in man. Acta Unio Int Contra Cancrum. 1953;9:531–541. 13124110

[44] Ezzati M, Hoorn SV, Rodgers A, Lopez AD, Mathers CD, Murray CJ. Comparative Risk Assessment Collaborating Group. Estimates of global and regional potential health gains from reducing multiple major risk factors. Lancet. 2003;362:271–280. 12892956 10.1016/s0140-6736(03)13968-2

[45] Brasil. Ministério da Saúde. Instituto Nacional de Câncer. Estatísticas de Câncer. Registros de Câncer de Base Populacional. [Cancer Statistics: Population Based Cancer Registries]. Rio de Janeiro: INCA; 2014 Available: http://www2.inca.gov.br/wps/wcm/connect/estatisticas/site/home/rcbp/

[46] Brasil. Ministério da Saúde. Sistema de Informações sobre Mortalidade. [Mortality Health System]. Brasília: Ministério da Saúde, 2014 Available: http://www2.datasus.gov.br/DATASUS/index.php?area=0205.

[47] Schottenfeld D, Beebe-Dimmer JL, Buffler PA, Omenn GS. Current perspective on the global and United States cancer burden attributable to lifestyle and environmental risk factors. Annu Rev Public Health. 2013;34:97–117. 23514316 10.1146/annurev-publhealth-031912-114350

[48] Whiteman DC, Webb PM, Green AC, Neale RE, Fritschi L, Bain CJ, et al Cancers in Australia in 2010 attributable to modifiable factors: summary and conclusions. Aust N Z J Public Health. 2015;39:477–484. 10.1111/1753-6405.12471 26437735 PMC4606779

[49] de Martel C, Ferlay J, Franceschi S, Vignat J, Bray F, Foman D, et al Global burden of cancers attributable to infections in 2008: a review and synthetic analysis. Lancet Oncol. 2012;13:607–615. 10.1016/S1470-2045(12)70137-7 22575588

[50] Valente VB, Covas DT, Passos ADC. Marcadores sorológicos das hepatites B e C em doadores desangue do Hemocentro de Ribeirão Preto, São Paulo. Rev Soc Bras Med Trop. 2005;38:488–492. 16410924 10.1590/s0037-86822005000600008

[51] Gomes FS, Lobstein T. Food and beverage transnational corporations and nutrition policy. UN SCN News. 2011;39:57–65.

[52] World Health Organization (WHO). Global Health Observatory. Overweight and obesity. Available: http://www.who.int/gho/ncd/risk_factors/overweight/en/

[53] Brenner DR. Cancer incidence due to excess body weight and leisure-time physical inactivity in Canada: implications for prevention. Prev Med. 2014;66:131–139. 10.1016/j.ypmed.2014.06.018 24967956

[54] Park S, Kim Y, Shin HR, Lee B, Shin A, Jung KW, et al Population-attributable causes of cancer in Korea: obesity and physical inactivity. PLOS One. 2014;9:e90871 10.1371/journal.pone.0090871 24722008 PMC3982956

[55] Milton K, Macniven R, Bauman A. Review of the epidemiological evidence for physical activity and health from low- and middle-income countries. Glob Public Health. 2014;9:369–381. 10.1080/17441692.2014.894548 24697197

[56] Victora CG, Barreto ML, do Carmo Leal M, Monteiro CA, Schmidt MI, Paim J, et al Health conditions and health-policy innovations in Brazil: the way forward. Lancet. 2011;377:2042–2053. 21561659 10.1016/S0140-6736(11)60055-X

[57] Purdue MP, Hutchings SJ, Rushton L, Silverman DT. The proportion of cancer attributable to occupational exposures. Ann Epidemiol. 2015;25:188–92. 25487971 10.1016/j.annepidem.2014.11.009 PMC4631263

[58] Santana VS, Ribeiro FS. Occupational cancer burden in developing countries and the problem of informal workers. Environ Health. 2011;10:S10 10.1186/1476-069X-10-S1-S10 21489206 PMC3073188

[59] Stahel A, Weder W, Lievens Y, Felip E, ESMO Guidelines Working Group. Malignant pleural mesothelioma: ESMO Clinical Practice Guidelines for diagnosis, treatment and follow-up. Ann Oncol. 2010;21:126–128. 20555061 10.1093/annonc/mdq173

[60] Bray F, Jemal A, Grey N, Ferlay J, Forman D. Global cancer transitions according to the Human Development Index (2008–2030): a population-based study. Lancet Oncol. 2012;13:790–801. 10.1016/S1470-2045(12)70211-5 22658655

[61] Malta DC, Morais Neto OL, Silva JB Junior. Apresentação do plano de ações estratégicas para o enfrentamento das doenças crônicas não transmissíveis no Brasil, 2011 a 2022. Epidemiol Serv Saúde. 2011;20:425–438. 10.5123/S1679-49742016000200016 27869955

