# Supplementary material for: The Fraction of Cancer Attributable to Ways of Life, Infections, Occupation, and Environmental Agents in Brazil in 2020
Source: PLoS One. 2016 Feb 10;11(2):e0148761. doi: 10.1371/journal.pone.0148761 (PMC4749327; doi:10.1371/journal.pone.0148761)
Supplement: S1 Table — (DOC) [file pone.0148761.s001.doc]

**S1 Table. Risk factors, relative risks, prevalence of exposures and specific and combined population attributable fractions by cancer sites among men**

| **Type of Cancer (CID-10)** | **Risk factor** | **Relative risk** | **Reference** | **Prevalence (%)** | **Reference** | **PAF* (%)** | **Combined PAF (%)** |
| --- | --- | --- | --- | --- | --- | --- | --- |
| **Oral cavity (C00-C08)** | Tobacco | 3.50 | Ferreira Antunes et al.,2013. | 24.50 | PETab, 2008 | 38.02 | 95.31 |
|  | Acohol | 5.49 | Turati et al., 2013. | 4.50 | VIGITEL, 2008 | 16.81 |  |
|  | Fruit consumption lower than 160g/daily | 1.96 | Pavia et al., 2006. | 87.8g/d** | POF, 2008 | 45.50 |  |
|  | Vegetable consumption lower than 240g/daily | 2.00 | Pavia et al., 2006. | 46.3g/d** | POF, 2008 | 81.33 |  |
|  | HPV | 2.00 | Hobbs et al., 2006. | 11.80 | Giuliano et al., 2008. | 10.55 |  |
| **Stomach (C16) (non- cardia portion)** | H Pylori | 2.56 | Cavaleiro-Pinto et al., 2011. | 84.0 | Rodrigues et al., 2005 | 56.72 | 87.96 |
|  | Fruit consumption lower than 160g/daily | 1.35 | Riboli and Norat, 2003. | 87.8g/d** | POF, 2008 | 19.54 |  |
|  | Vegetable consumption lower than 240g/daily | 1.23 | Riboli and Norat, 2003. | 46.3g/d** | POF, 2008 | 33.51 |  |
|  | Processed meat consumption above zero | 1.15 | Larsson et al., 2006. | 15.9g/d** | POF, 2008 | 7.14 |  |
|  | Salt Intake | 1.00 | Shikata et al., 2006. | 53.7 | POF, 2008 | 37.07 |  |
|  | Alcohol use | 1.17 | Tramacere et al., 2012. | 4.5 | VIGITEL*, 2008* | 0.76 |  |
|  | Tobacco | 1.47 | Bonequi et al., 2013. | 24.50 | PETab, 2008 | 10.34 |  |
| **Stomach (C16) (cardia portion)** | Fruit consumption lower than 160g/daily | 1.35 | Riboli and Norat, 2003. | 87.8g/d** | POF, 2008 | 19.54 | 65.45 |
|  | Vegetable consumption lower than 240g/daily | 1.23 | Riboli and Norat, 2003. | 46.3g/d** | POF, 2008 | 33.51 |  |
|  | Processed meat consumption above zero | 1.15 | Larsson et al., 2006. | 15.9g/d** | POF, 2008 | 7.14 |  |
|  | Salt Intake | 1.00 | Shikata et al., 2006. | 53.7 | POF, 2008 | 37.07 |  |
|  | Tobacco | 1.47 | Bonequi et al., 2013. | 24.50 | PETab, 2008 | 10.34 |  |
| **Esophagus (C15) (squamous)** | Tobacco | 4.10 | Castellsague et al., 1999. | 24.50 | PETab, 2008 | 49.21 | 72.84 |
|  | Fruit consumption lower than 160g/daily | 1.39 | Pavia et al., 2006 | 87.8g/d** | POF, 2008 | 21.12 |  |
|  | Vegetable consumption lower than 240g/daily | 1.12 | Pavia et al., 2006. | 46.3g/d** | POF, 2008 | 20.21 |  |
|  | Alcohol use | 4.89 | Islami al, 2011. | 4.50 | VIGITEL, 2008 | 14.89 |  |
|  | Occupational excposure to Gamma radiation | 2.65 | Wang et al., 2002. | 0.13 | CENSO, 2000 & PNAD, 2003 | 0.21 |  |
| **Esophagus (C15) (adenocarcinoma)** | Tobacco | 1.96 | Cook et al., 2010. | 24.50 | PETab, 2008 | 19.06 | 44.20 |
|  | Overweight/obesity (BMI kg/m^2^) |  | Harriss et al., 2009. |  | POF, 2008 | 31.06 |  |
|  | <25 | 1.00 |  | 44.46 |  |  |  |
|  | 25.0-29.9 | 1.52 |  | 41.02 |  |  |  |
|  | 30.0-34.9 | 2.31 |  | 11.59 |  |  |  |
|  | 35.0-39.9 | 3.51 |  | 2.30 |  |  |  |
|  | 40.0-44.9 | 5.34 |  | 0.41 |  |  |  |
|  | ≥45 | 8.11 |  | 0.04 |  |  |  |
| **Colon and rectum (C18-20)** | Alcohol use | 1.62 | Ferdirko et al., 2011. | 4.50 | VIGITEL, 2008 | 2.71 | 43.89 |
|  | Vegetable consumption lower than 240g/daily | 1.04 | Chan, 2011. | 46.3g/d** | POF, 2008 | 7.60 |  |
|  | Processed meat consumption above zero | 1.24 | Chan, 2011. | 15.9g/d** | POF, 2008 | 6.61 |  |
|  | Red meat consumption above 70g/daily | 1.17 | Chan, 2011. | 89.9g/d** | POF, 2008 | 3.08 |  |
|  | Physical inactivity | 1.32 | Wolin et al., 2009. | 28.70 | PNAD, 2008 | 18.13 |  |
|  | Overweight/obesity (BMI kg/m^2^) |  | Harriss et al., 2009. |  | POF, 2008 | 15.77 |  |
|  | <25 | 1.00 |  | 44.64 |  |  |  |
|  | 25.0-29.9 | 1.24 |  | 41.02 |  |  |  |
|  | 30.0-34.9 | 1.54 |  | 11.59 |  |  |  |
|  | 35.0-39.9 | 1.91 |  | 2.30 |  |  |  |
|  | ≥40 | 2.36 |  | 0.41 |  |  |  |
| **Liver (C22)** | Tobacco | 1.24 | Lee et al.,2009. | 24.50 | PETab, 2008 | 5.56 | 25.91 |
|  | Alcohol use | 1.16 | Lee et al.,2009. | 4.50 | VIGITEL, 2008 | 0.71 |  |
|  | HBV | 13.90 | Cho et al., 2011. | 60.0 | INH, 2010 | 7.18 |  |
|  | HCV | 12.29 | Cho et al., 2011. | 1.56 | INH, 2010 | 14.87 |  |
| **Pancreas (C25)** | Tobacco | 1.40 | Bosetti et al., 2012. | 25.0 | PETab, 2008 | 8.93 | 33.90 |
|  | Overweight/obesity (BMI kg/m^2^) |  | Aune et al., 2012. |  | POF, 2008 |  |  |
|  | <25 | 1.00 |  | 44.64 |  | 7.02 |  |
|  | 25.0-29.9 | 1.10 |  | 41.02 |  |  |  |
|  | 30.0-34.9 | 1.21 |  | 11.59 |  |  |  |
|  | 35.0-39.9 | 1.33 |  | 2.30 |  |  |  |
|  | 40.0-44.9 | 1.46 |  | 0.41 |  |  |  |
|  | ≥45 | 1.61 |  | 0.04 |  |  |  |
|  | Physical inactivity | 1.39 | O’Rorke et al., 2010. | 28.70 | PNAD, 2008 | 22.32 |  |
| **Gallbladder and bile ducts (C23-24)** | Overweight/obesity (BM kg/m^2^) |  | Larsson and Wolk, 2007. |  | POF, 2008 | 6.60 | 6.60 |
|  | <25 | 1.00 |  | 44.64 |  |  |  |
|  | 25.0-29.9 | 1.05 |  | 41.02 |  |  |  |
|  | ≥30 | 1.35 |  | 14.34 |  |  |  |
| **Oral pharynx (C09-10, C12-C14)** | HPV | 4.30 | Hobbs et al., 2006. | 12.8 | Giuliano et al.., 2008 | 28.03 | 28.03 |
| **Nasopharynx (C11)** | EBV | 16.00 | Hsu et al., 2009. | 54.1 | Figueira-Silva and Pereira, 2004 | 89.1 | 89.24 |
|  | Formaldehyde | 1.30 | Collins et al., 19972. | 4.18 | CENSO, 2000; PNAD, 2003 | 1.12 |  |
| **Sinonasal (C30-31)** | Wood dust | 4.2 | Pesche et al., 2008 | 5.33 | CENSO, 2000; PNAD, 2003 | 13.79 | 33.69 |
|  | Leather dust | 6.80 | Battista et al., 1995. | 1.55 |  | 8.26 |  |
|  | Nickel | 18.00 | **Binazzi et al., 2015** | 1.13 |  | 16.15 |  |
| **Larynx (C32)** | Tobacco | 8.33 | Wyss et al., 2013. | 25.0 | PETab, 2008 | 64.27 | 80.28 |
|  | Alcohol use | 2.62 | Islami et al., 2010. | 4.5 | VIGITEL, 2008 | 6.79 |  |
|  | Fruit consumption lower than 160g/daily | 1.09 | Pavia et al., 2006. | 87.8g/d** | POF, 2008 | 20.33 |  |
|  | Vegetable consumption lower than 240g/daily | 1.37 | Pavia et al., 2006. | 46.3g/d** | POF, 2008 | 14.91 |  |
|  | HPV | 2.00 | Hobbs et al., 2006. | 11.8 | Giuliano et al., 2008. | 10.55 |  |
|  | Asbestos | 1.44 | IOM, 2006. | 6.0 | CENSO, 2000; PNAD, 2003 | 2.34 |  |
| **Lung (C33-34)** | Tobacco | 21.5 | Thun et al., 2000. | 24.50 | PETab, 2008 | 83.28 | 88.74 |
|  | Fruit consumption lower than 160g/daily | 1.06 | W Cancer Res Fund, 2007. | 87.8g/d** | POF, 2008 | 5.43 |  |
|  | Vegetable consumption lower than 240g/daily | 1.05 | W Cancer Res Fund, 2007. | 46.3g/d** | POF, 2008 | 11.68 |  |
|  | Painting | 1.95 | Guha et al., 2010. | 4.44 | CENSO, 2000; PNAD, 2003 | 4.05 |  |
|  | Silica | 1.32 | Kurihara & Wada, 2004. | 6.48 | CENSO, 2000; PNAD, 2003 | 2.03 |  |
|  | Iron/steel | 1.40 | Bosetti et al., 2007. | 0.20 | CENSO, 2000; PNAD, 2003 | 0.08 |  |
|  | Benzopyrene | 1.20 | Armstrong et al., 2004. | 1.29 | CENSO, 2000; PNAD, 2003 | 0.002 |  |
|  | Nickel | 3.80 | Grimsrud et al., 2002. | 1.13 | CENSO, 2000; PNAD, 2003 | 3.07 |  |
|  | Diesel | 1,33 | Lipsett & Campleman, 1999. | 3.27 | CENSO, 2000; PNAD, 2003 | 1.06 |  |
|  | Asbestos | 1.66 | Lenters et al., 2011. | 6.00 | CENSO, 2000; PNAD, 2003 | 3.81 |  |
|  | Occupational exposure to Radon | 1.08 | Darby et al., 2006. | 6.00 | CENSO, 2000; PNAD, 2003 | 0.29 |  |
|  | Occupational exposure to Gamma radiation | 1.20 | Wang et al., 2002. | 0.13 | CENSO, 2000; PNAD, 2003 | 0.03 |  |
|  | Second-hand smoke | 1.31 | Kim et al., 2014. | 17.0 | PETab, 2008 | 5.01 |  |
|  | Particulate matter (PM10) | 1.08 | Hamra et al., 2014. | 39.0 µg/m^3***^ | Freitas et al., 2013. | 5.08 |  |
| **Cutaneous melanoma (C43)** | Environmental solar radiation | 2.03 | Gandini et al., 2005. | 39.62 | PNAD, 2008; Szklo et al.., 2007; Bakos et al.., 2013 | 27.18 | 28.20 |
|  | Occupational solar radiation | 2.03 | Gandini et al. 2005. |  | CENSO, 2000; PNAD, 2003 | 1.36 |  |
| **Prostate (C61)** | Physical inactivity | 1.11 | Liu et al., 2011. | 28.70 | PNAD, 2008 | 6.40 | 6.40 |
| **Penis (C60)** | HPV | 1.90 | Daling et al., 2005. | 11.80 | Giuliano et al., 2008. | 9.6 | 9.60 |
| **Non-Hodgkin lymphoma (C82-C85, C96)** | EBV | 5.59 | Goldacre et al., 2009. | 54.10 | Figueira-Silva & Pereira, 2004 | 70.85 | 71.51 |
|  | Rubber industry | 3.60 | Kogevinas et al., 1998. | 0.89 | CENSO 2000; PNAD 2003 | 2.27 |  |
| **Hodgkin lymphoma (C81)** | EBV | 4.00 | Hjalgrim et al., 2003. | 54.1 | Figueira-Silva & Pereira, 2004 | 61.83 | 61.83 |
| **Leukemia (C91-95)** | Rubber industry | 1.50 | Weiland et al., 1998. | 0.89 | CENSO, 2000; PNAD, 2003 | 0.44 | 16.79 |
|  | Benzene | 2.62 | Khalade et al., 2010. | 9.30 |  | 13.09 |  |
|  | Formaldehyde | 1.90 | Zhang et al., 2009. | 4.18 |  | 3.62 |  |
|  | Occupational exposure to Gamma radiation | 2.17 | Wang et al., 2002. | 0.13 |  | 0.15 |  |
| **Kidney (C64)** | Tobacco | 1.4 | Behrens and Leitzmann, 2013. | 24.50 | VIGITEL, 2008 | 8.94 | 35.60 |
|  | Overweight/obesity (BMI kg/m^2^) |  | Renehan et al., 2008. |  | POF, 2008 | 15.77 |  |
|  | <25 | 1.00 |  | 44.64 |  |  |  |
|  | 25.0-29.9 | 1.24 |  | 41.02 |  |  |  |
|  | 30.0-34.9 | 1.54 |  | 11.59 |  |  |  |
|  | 35.0-39.9 | 1.91 |  | 2.30 |  |  |  |
|  | ≥40 | 2.36 |  | 0.41 |  |  |  |
|  | Physical inactivity | 1.28 | Behrens & Leitzmann, 2013. | 28.70 | PNAD, 2008 | 16.19 |  |
| **Bladder (C67)** | Tobacco | 1.81 | Hemelt et al., 2009. | 24.50 | PETab, 2008 | 16.58 | 23.61 |
|  | Painting | 1.81 | Guha et al., 2010. | 4.44 | CENSO, 2000; PNAD, 2003 | 3.47 |  |
|  | Rubber industry | 5.20 | Kogevinas et al., 1988. | 0.89 | CENSO, 2000; PNAD, 2003 | 3.61 |  |
|  | Iron/steel | 1.29 | Bosetti et al., 2007. | 0,20 | CENSO, 2000; PNAD, 2003 | 0.59 |  |
|  | Diesel | 1.44 | Boffetta e Silverman, 2011 | 3.27 | CENSO, 2000; PNAD, 2003 | 1.41 |  |
|  | Occupational exposure to gamma radiation | 1.84 | Wang et al., 2002. | 0.13 | CENSO, 2000; PNAD, 2003 | 0.11 |  |
| **Mesothelioma (C45)** | Asbestos | 9.1 | Howel et al., 1997. | 6.00 | CENSO, 2000; PNAD, 2003 | 32.71 | 32.71 |

*PAF: Population attributable risk

**Average consumption

***Average annual exposure in metropolitan areas (34% of Brazilian population lives in metropolitan areas).

**References**

Armstrong B, Hutchinson E, Unwin J, Fletcher T. [Lung cancer risk after exposure to polycyclic aromatic hydrocarbons: a review and meta-analysis.](http://www.ncbi.nlm.nih.gov/pubmed/15198916) *Environ Health Perspect*. 2004;112:970-8.

[Aune D](http://www.ncbi.nlm.nih.gov/pubmed/?term=Aune D%5BAuthor%5D&cauthor=true&cauthor_uid=21890910), [Greenwood DC](http://www.ncbi.nlm.nih.gov/pubmed/?term=Greenwood DC%5BAuthor%5D&cauthor=true&cauthor_uid=21890910), [Chan DS](http://www.ncbi.nlm.nih.gov/pubmed/?term=Chan DS%5BAuthor%5D&cauthor=true&cauthor_uid=21890910), [Vieira R](http://www.ncbi.nlm.nih.gov/pubmed/?term=Vieira R%5BAuthor%5D&cauthor=true&cauthor_uid=21890910), [Vieira AR](http://www.ncbi.nlm.nih.gov/pubmed/?term=Vieira AR%5BAuthor%5D&cauthor=true&cauthor_uid=21890910), [Navarro Rosenblatt DA](http://www.ncbi.nlm.nih.gov/pubmed/?term=Navarro Rosenblatt DA%5BAuthor%5D&cauthor=true&cauthor_uid=21890910), [Cade JE](http://www.ncbi.nlm.nih.gov/pubmed/?term=Cade JE%5BAuthor%5D&cauthor=true&cauthor_uid=21890910), [Burley VJ](http://www.ncbi.nlm.nih.gov/pubmed/?term=Burley VJ%5BAuthor%5D&cauthor=true&cauthor_uid=21890910), [Norat T](http://www.ncbi.nlm.nih.gov/pubmed/?term=Norat T%5BAuthor%5D&cauthor=true&cauthor_uid=21890910). Body mass index, abdominal fatness and pancreatic cancer risk: a systematic review and non-linear dose-response meta-analysis of prospective studies. [*Ann Oncol.*](http://www.ncbi.nlm.nih.gov/pubmed/?term=Aune+et+al.+Annals+of+Oncology+2012%3B+23%3A+843–852) 2012;23:843-52.

Battista G, Comba P, Orsi D, Norpoth K, Maier A. [Nasal cancer in leather workers: an occupational disease.](http://www.ncbi.nlm.nih.gov/pubmed/7860613) *J Cancer Res Clin Oncol.* 1995;121:1-6.

Behrens G, Leitzmann MF. The association between physical activity and renal cancer: systematic review and meta-analysis. *Br J Cancer.* 2013;108:798-811.

Binazzi A, Ferrante P, Marinaccio A. [Occupational exposure and sinonasal cancer: a systematic review and meta-analysis.](http://www.ncbi.nlm.nih.gov/pubmed/25885319) *BMC Cancer*. 2015; 15:49

[Boffetta P](http://www.ncbi.nlm.nih.gov/pubmed/?term=Boffetta P%5BAuthor%5D&cauthor=true&cauthor_uid=11138807), [Silverman DT](http://www.ncbi.nlm.nih.gov/pubmed/?term=Silverman DT%5BAuthor%5D&cauthor=true&cauthor_uid=11138807). A meta-analysis of bladder cancer and diesel exhaust exposure. [*Epidemiology.*](http://www.ncbi.nlm.nih.gov/pubmed/11138807) 2001;12:125-30.

[Bonequi P](http://www.ncbi.nlm.nih.gov/pubmed?term=Bonequi P%5BAuthor%5D&cauthor=true&cauthor_uid=23224270), [Meneses-González F](http://www.ncbi.nlm.nih.gov/pubmed?term=Meneses-González F%5BAuthor%5D&cauthor=true&cauthor_uid=23224270), [Correa P](http://www.ncbi.nlm.nih.gov/pubmed?term=Correa P%5BAuthor%5D&cauthor=true&cauthor_uid=23224270), [Rabkin CS](http://www.ncbi.nlm.nih.gov/pubmed?term=Rabkin CS%5BAuthor%5D&cauthor=true&cauthor_uid=23224270), [Camargo MC](http://www.ncbi.nlm.nih.gov/pubmed?term=Camargo MC%5BAuthor%5D&cauthor=true&cauthor_uid=23224270). Risk factors for gastric cancer in Latin America: a meta-analysis. [*Cancer Causes Control.*](http://www.ncbi.nlm.nih.gov/pubmed/23224270) 2013;24:217-31.

Bosetti C, Boffetta P, La Vecchia C. [Occupational exposures to polycyclic aromatic hydrocarbons, and respiratory and urinary tract cancers: a quantitative review to 2005.](http://www.ncbi.nlm.nih.gov/pubmed/16936186) *Ann Oncol*. 2007;18:431-46.

[Bosetti C](http://www.ncbi.nlm.nih.gov/pubmed?term=Bosetti C%5BAuthor%5D&cauthor=true&cauthor_uid=22104574), [Lucenteforte E](http://www.ncbi.nlm.nih.gov/pubmed?term=Lucenteforte E%5BAuthor%5D&cauthor=true&cauthor_uid=22104574), [Silverman DT](http://www.ncbi.nlm.nih.gov/pubmed?term=Silverman DT%5BAuthor%5D&cauthor=true&cauthor_uid=22104574), [Petersen G](http://www.ncbi.nlm.nih.gov/pubmed?term=Petersen G%5BAuthor%5D&cauthor=true&cauthor_uid=22104574), [Bracci PM](http://www.ncbi.nlm.nih.gov/pubmed?term=Bracci PM%5BAuthor%5D&cauthor=true&cauthor_uid=22104574), [Ji BT](http://www.ncbi.nlm.nih.gov/pubmed?term=Ji BT%5BAuthor%5D&cauthor=true&cauthor_uid=22104574), [Negri E](http://www.ncbi.nlm.nih.gov/pubmed?term=Negri E%5BAuthor%5D&cauthor=true&cauthor_uid=22104574), [Li D](http://www.ncbi.nlm.nih.gov/pubmed?term=Li D%5BAuthor%5D&cauthor=true&cauthor_uid=22104574), [Risch HA](http://www.ncbi.nlm.nih.gov/pubmed?term=Risch HA%5BAuthor%5D&cauthor=true&cauthor_uid=22104574), [Olson SH](http://www.ncbi.nlm.nih.gov/pubmed?term=Olson SH%5BAuthor%5D&cauthor=true&cauthor_uid=22104574), [Gallinger S](http://www.ncbi.nlm.nih.gov/pubmed?term=Gallinger S%5BAuthor%5D&cauthor=true&cauthor_uid=22104574), [Miller AB](http://www.ncbi.nlm.nih.gov/pubmed?term=Miller AB%5BAuthor%5D&cauthor=true&cauthor_uid=22104574), [Bueno-de-Mesquita HB](http://www.ncbi.nlm.nih.gov/pubmed?term=Bueno-de-Mesquita HB%5BAuthor%5D&cauthor=true&cauthor_uid=22104574),[Talamini R](http://www.ncbi.nlm.nih.gov/pubmed?term=Talamini R%5BAuthor%5D&cauthor=true&cauthor_uid=22104574), *et al.*.. Cigarette smoking and pancreatic cancer: an analysis from the International Pancreatic Cancer Case-Control Consortium (Panc4). [*Ann Oncol.*](http://www.ncbi.nlm.nih.gov/pubmed/22104574) 2012;23:1880-8.

Camargo MC, Stayner LT, Straif K, Reina M, Al-Alem U, Demers PA, Landrigan PJ. [Occupational exposure to asbestos and ovarian cancer: a meta-analysis.](http://www.ncbi.nlm.nih.gov/pubmed/21642044) *Environ Health Perspect*. 2011;119:1211-7.

[Castellsagué X](http://www.ncbi.nlm.nih.gov/pubmed?term=Castellsagué X%5BAuthor%5D&cauthor=true&cauthor_uid=10417762), [Muñoz N](http://www.ncbi.nlm.nih.gov/pubmed?term=Muñoz N%5BAuthor%5D&cauthor=true&cauthor_uid=10417762), [De Stefani E](http://www.ncbi.nlm.nih.gov/pubmed?term=De Stefani E%5BAuthor%5D&cauthor=true&cauthor_uid=10417762), [Victora CG](http://www.ncbi.nlm.nih.gov/pubmed?term=Victora CG%5BAuthor%5D&cauthor=true&cauthor_uid=10417762), [Castelletto R](http://www.ncbi.nlm.nih.gov/pubmed?term=Castelletto R%5BAuthor%5D&cauthor=true&cauthor_uid=10417762), [Rolón PA](http://www.ncbi.nlm.nih.gov/pubmed?term=Rolón PA%5BAuthor%5D&cauthor=true&cauthor_uid=10417762), [Quintana MJ](http://www.ncbi.nlm.nih.gov/pubmed?term=Quintana MJ%5BAuthor%5D&cauthor=true&cauthor_uid=10417762). Independent and joint effects of tobacco smoking and alcohol drinking on the risk of esophageal cancer in men and women. [*Int J Cancer.*](http://www.ncbi.nlm.nih.gov/pubmed/10417762)1999;82:657-64.

[Cavaleiro-Pinto](http://link.springer.com/search?facet-author="Marlene+Cavaleiro-Pinto") M, [Peleteiro](http://link.springer.com/search?facet-author="Bárbara+Peleteiro") B, [Lunet](http://link.springer.com/search?facet-author="Nuno+Lunet") N, [Barros](http://link.springer.com/search?facet-author="Henrique+Barros") H. Helicobacter pylori infection and gastric cardia cancer: systematic review and meta-analysis. [*Cancer Causes and Control*](http://link.springer.com/journal/10552) 2011;22:375-87.

CCGESOC. [Collaborative Group on Epidemiological Studies of Ovarian Cancer](http://www.ncbi.nlm.nih.gov/pubmed/?term=Collaborative Group on Epidemiological Studies of Ovarian Cancer%5BCorporate Author%5D), [Beral V](http://www.ncbi.nlm.nih.gov/pubmed/?term=Beral V%5BAuthor%5D&cauthor=true&cauthor_uid=22863523), [Gaitskell K](http://www.ncbi.nlm.nih.gov/pubmed/?term=Gaitskell K%5BAuthor%5D&cauthor=true&cauthor_uid=22863523), [Hermon C](http://www.ncbi.nlm.nih.gov/pubmed/?term=Hermon C%5BAuthor%5D&cauthor=true&cauthor_uid=22863523), [Moser K](http://www.ncbi.nlm.nih.gov/pubmed/?term=Moser K%5BAuthor%5D&cauthor=true&cauthor_uid=22863523), [Reeves G](http://www.ncbi.nlm.nih.gov/pubmed/?term=Reeves G%5BAuthor%5D&cauthor=true&cauthor_uid=22863523), [Peto R](http://www.ncbi.nlm.nih.gov/pubmed/?term=Peto R%5BAuthor%5D&cauthor=true&cauthor_uid=22863523). Ovarian cancer and smoking: individual participant meta-analysis including 28,114 women with ovarian cancer from 51 epidemiological studies. [*Lancet Oncol.*](http://www.ncbi.nlm.nih.gov/pubmed/?term=lancet+Oncol.+2012+Sep%3B13(9)%3A946-56.) 2012;13:946-56.

CENSO 2000. *Censo Demográfico 2000. Pessoas ocupadas por atividade no Brasil e grandes regiõ*es. Instituto Brasileiro de Geografia e Estatística (IBGE).. Rio de Janeiro: IBGE, 2001.

<http://www.ibge.gov.br/home/estatistica/populacao/censo2000/default_populacao.shtm>

[Chan DS](http://www.ncbi.nlm.nih.gov/pubmed/?term=Chan DS%5BAuthor%5D&cauthor=true&cauthor_uid=21674008), [Lau R](http://www.ncbi.nlm.nih.gov/pubmed/?term=Lau R%5BAuthor%5D&cauthor=true&cauthor_uid=21674008), [Aune D](http://www.ncbi.nlm.nih.gov/pubmed/?term=Aune D%5BAuthor%5D&cauthor=true&cauthor_uid=21674008), [Vieira R](http://www.ncbi.nlm.nih.gov/pubmed/?term=Vieira R%5BAuthor%5D&cauthor=true&cauthor_uid=21674008), [Greenwood DC](http://www.ncbi.nlm.nih.gov/pubmed/?term=Greenwood DC%5BAuthor%5D&cauthor=true&cauthor_uid=21674008), [Kampman E](http://www.ncbi.nlm.nih.gov/pubmed/?term=Kampman E%5BAuthor%5D&cauthor=true&cauthor_uid=21674008), [Norat T](http://www.ncbi.nlm.nih.gov/pubmed/?term=Norat T%5BAuthor%5D&cauthor=true&cauthor_uid=21674008). Red and processed meat and colorectal cancer incidence: meta-analysis of prospective studies. [*PLoS One.*](http://www.ncbi.nlm.nih.gov/pubmed/?term=Chan+et+al.+PLoS+One.+2011%3B6(6)%3Ae20456) 2011;6:e20456.

[Cho LY](http://www.ncbi.nlm.nih.gov/pubmed/?term=Cho LY%5BAuthor%5D&cauthor=true&cauthor_uid=20232388), [Yang JJ](http://www.ncbi.nlm.nih.gov/pubmed/?term=Yang JJ%5BAuthor%5D&cauthor=true&cauthor_uid=20232388), [Ko KP](http://www.ncbi.nlm.nih.gov/pubmed/?term=Ko KP%5BAuthor%5D&cauthor=true&cauthor_uid=20232388), [Park B](http://www.ncbi.nlm.nih.gov/pubmed/?term=Park B%5BAuthor%5D&cauthor=true&cauthor_uid=20232388), [Shin A](http://www.ncbi.nlm.nih.gov/pubmed/?term=Shin A%5BAuthor%5D&cauthor=true&cauthor_uid=20232388), [Lim MK](http://www.ncbi.nlm.nih.gov/pubmed/?term=Lim MK%5BAuthor%5D&cauthor=true&cauthor_uid=20232388), [Oh JK](http://www.ncbi.nlm.nih.gov/pubmed/?term=Oh JK%5BAuthor%5D&cauthor=true&cauthor_uid=20232388), [Park S](http://www.ncbi.nlm.nih.gov/pubmed/?term=Park S%5BAuthor%5D&cauthor=true&cauthor_uid=20232388), [Kim YJ](http://www.ncbi.nlm.nih.gov/pubmed/?term=Kim YJ%5BAuthor%5D&cauthor=true&cauthor_uid=20232388), [Shin HR](http://www.ncbi.nlm.nih.gov/pubmed/?term=Shin HR%5BAuthor%5D&cauthor=true&cauthor_uid=20232388), [Yoo KY](http://www.ncbi.nlm.nih.gov/pubmed/?term=Yoo KY%5BAuthor%5D&cauthor=true&cauthor_uid=20232388), [Park SK](http://www.ncbi.nlm.nih.gov/pubmed/?term=Park SK%5BAuthor%5D&cauthor=true&cauthor_uid=20232388). Coinfection of hepatitis B and C viruses and risk of hepatocellular carcinoma: systematic review and meta-analysis. [*Int J Cancer.*](http://www.ncbi.nlm.nih.gov/pubmed/?term=6.%09Cho+LY%2CYang+JJ%2CKwang-Pil+KO%2C+Boyoung+P%2CAesun+S%2CMin+KL+et+al.Coinfection+of+hepatitis+B+and+C+viruses+and+risk+of+hepatocellular+carcinoma%3A+systematic+review+and+meta-anlysis.) 2011;128:176-84.

[Collins JJ](http://www.ncbi.nlm.nih.gov/pubmed?term=Collins JJ%5BAuthor%5D&cauthor=true&cauthor_uid=9253725), [Acquavella JF](http://www.ncbi.nlm.nih.gov/pubmed?term=Acquavella JF%5BAuthor%5D&cauthor=true&cauthor_uid=9253725), [Esmen NA](http://www.ncbi.nlm.nih.gov/pubmed?term=Esmen NA%5BAuthor%5D&cauthor=true&cauthor_uid=9253725). An updated meta-analysis of formaldehyde exposure and upper respiratory tract cancers. [*J Occup Environ Med*.](http://www.ncbi.nlm.nih.gov/pubmed/?term=Collins+AND+cancer+AND+formaldeyde+1997) 1997;39:639-51.

[Cook MB](http://www.ncbi.nlm.nih.gov/pubmed?term=Cook MB%5BAuthor%5D&cauthor=true&cauthor_uid=20716718), [Kamangar F](http://www.ncbi.nlm.nih.gov/pubmed?term=Kamangar F%5BAuthor%5D&cauthor=true&cauthor_uid=20716718), [Whiteman DC](http://www.ncbi.nlm.nih.gov/pubmed?term=Whiteman DC%5BAuthor%5D&cauthor=true&cauthor_uid=20716718), [Freedman ND](http://www.ncbi.nlm.nih.gov/pubmed?term=Freedman ND%5BAuthor%5D&cauthor=true&cauthor_uid=20716718), [Gammon MD](http://www.ncbi.nlm.nih.gov/pubmed?term=Gammon MD%5BAuthor%5D&cauthor=true&cauthor_uid=20716718), [Bernstein L](http://www.ncbi.nlm.nih.gov/pubmed?term=Bernstein L%5BAuthor%5D&cauthor=true&cauthor_uid=20716718), [Brown LM](http://www.ncbi.nlm.nih.gov/pubmed?term=Brown LM%5BAuthor%5D&cauthor=true&cauthor_uid=20716718), [Risch HA](http://www.ncbi.nlm.nih.gov/pubmed?term=Risch HA%5BAuthor%5D&cauthor=true&cauthor_uid=20716718), [Ye W](http://www.ncbi.nlm.nih.gov/pubmed?term=Ye W%5BAuthor%5D&cauthor=true&cauthor_uid=20716718), [Sharp L](http://www.ncbi.nlm.nih.gov/pubmed?term=Sharp L%5BAuthor%5D&cauthor=true&cauthor_uid=20716718), [Pandeya N](http://www.ncbi.nlm.nih.gov/pubmed?term=Pandeya N%5BAuthor%5D&cauthor=true&cauthor_uid=20716718), [Webb PM](http://www.ncbi.nlm.nih.gov/pubmed?term=Webb PM%5BAuthor%5D&cauthor=true&cauthor_uid=20716718), [Wu AH](http://www.ncbi.nlm.nih.gov/pubmed?term=Wu AH%5BAuthor%5D&cauthor=true&cauthor_uid=20716718) et al... Cigarette smoking and adenocarcinomas of the esophagus and esophagogastric junction: a pooled analysis from the international BEACON consortium. *J Natl Cancer Inst*. 2010;102:1344-53.

[Daling JR](http://www.ncbi.nlm.nih.gov/pubmed?term=Daling JR%5BAuthor%5D&cauthor=true&cauthor_uid=15825185), [Madeleine MM](http://www.ncbi.nlm.nih.gov/pubmed?term=Madeleine MM%5BAuthor%5D&cauthor=true&cauthor_uid=15825185), [Johnson LG](http://www.ncbi.nlm.nih.gov/pubmed?term=Johnson LG%5BAuthor%5D&cauthor=true&cauthor_uid=15825185), [Schwartz SM](http://www.ncbi.nlm.nih.gov/pubmed?term=Schwartz SM%5BAuthor%5D&cauthor=true&cauthor_uid=15825185), [Shera KA](http://www.ncbi.nlm.nih.gov/pubmed?term=Shera KA%5BAuthor%5D&cauthor=true&cauthor_uid=15825185), [Wurscher MA](http://www.ncbi.nlm.nih.gov/pubmed?term=Wurscher MA%5BAuthor%5D&cauthor=true&cauthor_uid=15825185), [Carter JJ](http://www.ncbi.nlm.nih.gov/pubmed?term=Carter JJ%5BAuthor%5D&cauthor=true&cauthor_uid=15825185), [Porter PL](http://www.ncbi.nlm.nih.gov/pubmed?term=Porter PL%5BAuthor%5D&cauthor=true&cauthor_uid=15825185), [Galloway DA](http://www.ncbi.nlm.nih.gov/pubmed?term=Galloway DA%5BAuthor%5D&cauthor=true&cauthor_uid=15825185), [McDougall JK](http://www.ncbi.nlm.nih.gov/pubmed?term=McDougall JK%5BAuthor%5D&cauthor=true&cauthor_uid=15825185), [Krieger JN](http://www.ncbi.nlm.nih.gov/pubmed?term=Krieger JN%5BAuthor%5D&cauthor=true&cauthor_uid=15825185). Penile cancer: importance of circumcision, human papillomavirus and smoking in in situ and invasive disease. [*Int J Cancer.*](http://www.ncbi.nlm.nih.gov/pubmed/15825185) 2005;116:606-16.

Darby S, Hill D, Deo H, Auvinen A, Barros-Dios JM, Baysson H, Bochicchio F, Falk R, Farchi S, Figueiras A, Hakama M, Heid I, Hunter N et al... [Residential radon and lung cancer--detailed results of a collaborative analysis of individual data on 7148 persons with lung cancer and 14,208 persons without lungcancer from 13 epidemiologic studies in Europe.](http://www.ncbi.nlm.nih.gov/pubmed/16538937) *Scand J Work Environ Health*. 2006;32:1S-83S.

[Fedirko V](http://www.ncbi.nlm.nih.gov/pubmed?term=Fedirko V%5BAuthor%5D&cauthor=true&cauthor_uid=21307158), [Tramacere I](http://www.ncbi.nlm.nih.gov/pubmed?term=Tramacere I%5BAuthor%5D&cauthor=true&cauthor_uid=21307158), [Bagnardi V](http://www.ncbi.nlm.nih.gov/pubmed?term=Bagnardi V%5BAuthor%5D&cauthor=true&cauthor_uid=21307158), [Rota M](http://www.ncbi.nlm.nih.gov/pubmed?term=Rota M%5BAuthor%5D&cauthor=true&cauthor_uid=21307158), [Scotti L](http://www.ncbi.nlm.nih.gov/pubmed?term=Scotti L%5BAuthor%5D&cauthor=true&cauthor_uid=21307158), [Islami F](http://www.ncbi.nlm.nih.gov/pubmed?term=Islami F%5BAuthor%5D&cauthor=true&cauthor_uid=21307158), [Negri E](http://www.ncbi.nlm.nih.gov/pubmed?term=Negri E%5BAuthor%5D&cauthor=true&cauthor_uid=21307158), [Straif K](http://www.ncbi.nlm.nih.gov/pubmed?term=Straif K%5BAuthor%5D&cauthor=true&cauthor_uid=21307158), [Romieu I](http://www.ncbi.nlm.nih.gov/pubmed?term=Romieu I%5BAuthor%5D&cauthor=true&cauthor_uid=21307158), [La Vecchia C](http://www.ncbi.nlm.nih.gov/pubmed?term=La Vecchia C%5BAuthor%5D&cauthor=true&cauthor_uid=21307158), [Boffetta P](http://www.ncbi.nlm.nih.gov/pubmed?term=Boffetta P%5BAuthor%5D&cauthor=true&cauthor_uid=21307158), [Jenab M](http://www.ncbi.nlm.nih.gov/pubmed?term=Jenab M%5BAuthor%5D&cauthor=true&cauthor_uid=21307158). Alcohol drinking and colorectal cancer risk: an overall and dose-response meta-analysis of published studies. [*Ann Oncol.*](http://www.ncbi.nlm.nih.gov/pubmed/21307158) 2011;22:1958-72.

[Ferreira Antunes JL](http://www.ncbi.nlm.nih.gov/pubmed?term=Ferreira Antunes JL%5BAuthor%5D&cauthor=true&cauthor_uid=23874521), [Toporcov TN](http://www.ncbi.nlm.nih.gov/pubmed?term=Toporcov TN%5BAuthor%5D&cauthor=true&cauthor_uid=23874521), [Biazevic MG](http://www.ncbi.nlm.nih.gov/pubmed?term=Biazevic MG%5BAuthor%5D&cauthor=true&cauthor_uid=23874521), [Boing AF](http://www.ncbi.nlm.nih.gov/pubmed?term=Boing AF%5BAuthor%5D&cauthor=true&cauthor_uid=23874521), [Scully C](http://www.ncbi.nlm.nih.gov/pubmed?term=Scully C%5BAuthor%5D&cauthor=true&cauthor_uid=23874521), [Petti S](http://www.ncbi.nlm.nih.gov/pubmed?term=Petti S%5BAuthor%5D&cauthor=true&cauthor_uid=23874521). Joint and independent effects of alcohol drinking and tobacco smoking on oral cancer: a large case-control study. [*PLoS One.*](http://www.ncbi.nlm.nih.gov/pubmed/23874521) 2013;8:e68132.

Freitas CU, Junger W, de Leon AP, Silva MAFR, Gouveia N. Poluição do ar em cidades brasileiras: selecionando indicadores de impacto na saúde para fins de vigilância. *Epidemiol Serv Saúde*. 2013;22:445-54.

[Gandini S](http://www.ncbi.nlm.nih.gov/pubmed?term=Gandini S%5BAuthor%5D&cauthor=true&cauthor_uid=15617990), [Sera F](http://www.ncbi.nlm.nih.gov/pubmed?term=Sera F%5BAuthor%5D&cauthor=true&cauthor_uid=15617990), [Cattaruzza MS](http://www.ncbi.nlm.nih.gov/pubmed?term=Cattaruzza MS%5BAuthor%5D&cauthor=true&cauthor_uid=15617990), [Pasquini P](http://www.ncbi.nlm.nih.gov/pubmed?term=Pasquini P%5BAuthor%5D&cauthor=true&cauthor_uid=15617990), [Picconi O](http://www.ncbi.nlm.nih.gov/pubmed?term=Picconi O%5BAuthor%5D&cauthor=true&cauthor_uid=15617990), [Boyle P](http://www.ncbi.nlm.nih.gov/pubmed?term=Boyle P%5BAuthor%5D&cauthor=true&cauthor_uid=15617990), [Melchi CF](http://www.ncbi.nlm.nih.gov/pubmed?term=Melchi CF%5BAuthor%5D&cauthor=true&cauthor_uid=15617990). Meta-analysis of risk factors for cutaneous melanoma: II. Sun exposure. [*Eur J Cancer.*](http://www.ncbi.nlm.nih.gov/pubmed/15617990)2005;41:45-60.

Gierisch JM,RemyRC,Urrutia RP. Oral contraceptive use and risk of breast, cervical,colorectal and endometrial cancers: a systematic review. *Cancer Epidemol Biomarkers Prev*.2013;6:1-38.

[Girianelli VR,](http://pesquisa.bvsalud.org/ses/?lang=pt&q=au:"Girianelli, Vania Reis") [Thuler LCS,](http://pesquisa.bvsalud.org/ses/?lang=pt&q=au:"Thuler, Luiz Claudio Santos") Azevedo e [Silva G.](http://pesquisa.bvsalud.org/ses/?lang=pt&q=au:"Silva, Gulnar Azevedo e") Prevalência de HPV em mulheres assistidas pela estratégia saúde da família na Baixada Fluminense do Estado do Rio de Janeiro / Prevalence of HPV infection among women covered by the family health program in the Baixada Fluminense, Rio de Janeiro, Brazil. [*Rev. Bras.Ginecol.Obstet*](http://portal.revistas.bvs.br/transf.php?xsl=xsl/titles.xsl&xml=http://catserver.bireme.br/cgi-bin/wxis1660.exe/?IsisScript=../cgi-bin/catrevistas/catrevistas.xis|database_name=TITLES|list_type=title|cat_name=ALL|from=1|count=50&lang=pt&comefrom=home&home=false&task=show_magazines&request_made_adv_search=false&lang=pt&show_adv_search=false&help_file=/help_pt.htm&connector=ET&search_exp=Rev. bras. ginecol. obstet). 2011*;*32:39-46.

# [**Giuliano AR**](http://www.ncbi.nlm.nih.gov/pubmed/?term=Giuliano AR%5BAuthor%5D&cauthor=true&cauthor_uid=18708396), [**Lazcano-Ponce E**](http://www.ncbi.nlm.nih.gov/pubmed/?term=Lazcano-Ponce E%5BAuthor%5D&cauthor=true&cauthor_uid=18708396), [**Villa LL**](http://www.ncbi.nlm.nih.gov/pubmed/?term=Villa LL%5BAuthor%5D&cauthor=true&cauthor_uid=18708396), [**Flores R**](http://www.ncbi.nlm.nih.gov/pubmed/?term=Flores R%5BAuthor%5D&cauthor=true&cauthor_uid=18708396), [**Salmeron J**](http://www.ncbi.nlm.nih.gov/pubmed/?term=Salmeron J%5BAuthor%5D&cauthor=true&cauthor_uid=18708396), [**Lee JH**](http://www.ncbi.nlm.nih.gov/pubmed/?term=Lee JH%5BAuthor%5D&cauthor=true&cauthor_uid=18708396), [**Papenfuss MR**](http://www.ncbi.nlm.nih.gov/pubmed/?term=Papenfuss MR%5BAuthor%5D&cauthor=true&cauthor_uid=18708396), [**Abrahamsen M**](http://www.ncbi.nlm.nih.gov/pubmed/?term=Abrahamsen M%5BAuthor%5D&cauthor=true&cauthor_uid=18708396), [**Jolles E**](http://www.ncbi.nlm.nih.gov/pubmed/?term=Jolles E%5BAuthor%5D&cauthor=true&cauthor_uid=18708396), [**Nielson CM**](http://www.ncbi.nlm.nih.gov/pubmed/?term=Nielson CM%5BAuthor%5D&cauthor=true&cauthor_uid=18708396), [**Baggio ML**](http://www.ncbi.nlm.nih.gov/pubmed/?term=Baggio ML%5BAuthor%5D&cauthor=true&cauthor_uid=18708396), [**Silva R**](http://www.ncbi.nlm.nih.gov/pubmed/?term=Silva R%5BAuthor%5D&cauthor=true&cauthor_uid=18708396),[**Quiterio M**](http://www.ncbi.nlm.nih.gov/pubmed/?term=Quiterio M%5BAuthor%5D&cauthor=true&cauthor_uid=18708396). The human papillomavirus infection in men study: human papillomavirus prevalence and typedistribution among men residing in Brazil, Mexico, and the United States. [***Cancer Epidemiol Biomarkers Prev*.**](http://www.ncbi.nlm.nih.gov/pubmed/18708396)2008;17:2036-43.

Goldacre MJ, Wotton CJ, Yeates DGR. Associations between infectious mononucleosis and cancer: record-linkage studies. *Epidemiol. Infect.* 2009;137:672–680.

Grimsrud TK, Berge SR, Haldorsen T, Andersen A. [Exposure to different forms of nickel and risk of lung cancer.](http://www.ncbi.nlm.nih.gov/pubmed/12480657) *Am J Epidemiol.*  2002;156:1123-32.

Guha N, Merletti F, Steenland NK, Altieri A, Cogliano V, Straif K. [Lung cancer risk in painters: a meta-analysis.](http://www.ncbi.nlm.nih.gov/pubmed/20064777) *Environ Health Perspect.* 2010;118:303-12.

[Hamajima N](http://www.ncbi.nlm.nih.gov/pubmed?term=Hamajima N%5BAuthor%5D&cauthor=true&cauthor_uid=12439712), [Hirose K](http://www.ncbi.nlm.nih.gov/pubmed?term=Hirose K%5BAuthor%5D&cauthor=true&cauthor_uid=12439712), [Tajima K](http://www.ncbi.nlm.nih.gov/pubmed?term=Tajima K%5BAuthor%5D&cauthor=true&cauthor_uid=12439712), [Rohan T](http://www.ncbi.nlm.nih.gov/pubmed?term=Rohan T%5BAuthor%5D&cauthor=true&cauthor_uid=12439712), [Calle EE](http://www.ncbi.nlm.nih.gov/pubmed?term=Calle EE%5BAuthor%5D&cauthor=true&cauthor_uid=12439712), [Heath CW Jr](http://www.ncbi.nlm.nih.gov/pubmed?term=Heath CW Jr%5BAuthor%5D&cauthor=true&cauthor_uid=12439712), [Coates RJ](http://www.ncbi.nlm.nih.gov/pubmed?term=Coates RJ%5BAuthor%5D&cauthor=true&cauthor_uid=12439712), [Liff JM](http://www.ncbi.nlm.nih.gov/pubmed?term=Liff JM%5BAuthor%5D&cauthor=true&cauthor_uid=12439712), [Talamini R](http://www.ncbi.nlm.nih.gov/pubmed?term=Talamini R%5BAuthor%5D&cauthor=true&cauthor_uid=12439712), [Chantarakul N](http://www.ncbi.nlm.nih.gov/pubmed?term=Chantarakul N%5BAuthor%5D&cauthor=true&cauthor_uid=12439712), [Koetsawang S](http://www.ncbi.nlm.nih.gov/pubmed?term=Koetsawang S%5BAuthor%5D&cauthor=true&cauthor_uid=12439712), [Rachawat D](http://www.ncbi.nlm.nih.gov/pubmed?term=Rachawat D%5BAuthor%5D&cauthor=true&cauthor_uid=12439712), et al.. ; [Collaborative Group on Hormonal Factors in Breast Cancer](http://www.ncbi.nlm.nih.gov/pubmed?term=Collaborative Group on Hormonal Factors in Breast Cancer%5BCorporate Author%5D). Alcohol, tobacco and breast cancer--collaborative reanalysis of individual data from 53 epidemiological studies, including 58,515 women with breast cancer and 95,067 women without the disease. [*Br J Cancer*.](http://www.ncbi.nlm.nih.gov/pubmed/12439712) 2002;87:1234-45.

[Hamra GB](http://www.ncbi.nlm.nih.gov/pubmed?term=Hamra GB%5BAuthor%5D&cauthor=true&cauthor_uid=24911630), [Guha N](http://www.ncbi.nlm.nih.gov/pubmed?term=Guha N%5BAuthor%5D&cauthor=true&cauthor_uid=24911630), [Cohen A](http://www.ncbi.nlm.nih.gov/pubmed?term=Cohen A%5BAuthor%5D&cauthor=true&cauthor_uid=24911630), [Laden F](http://www.ncbi.nlm.nih.gov/pubmed?term=Laden F%5BAuthor%5D&cauthor=true&cauthor_uid=24911630), [Raaschou-Nielsen O](http://www.ncbi.nlm.nih.gov/pubmed?term=Raaschou-Nielsen O%5BAuthor%5D&cauthor=true&cauthor_uid=24911630), [Samet JM](http://www.ncbi.nlm.nih.gov/pubmed?term=Samet JM%5BAuthor%5D&cauthor=true&cauthor_uid=24911630), [Vineis P](http://www.ncbi.nlm.nih.gov/pubmed?term=Vineis P%5BAuthor%5D&cauthor=true&cauthor_uid=24911630), [Forastiere F](http://www.ncbi.nlm.nih.gov/pubmed?term=Forastiere F%5BAuthor%5D&cauthor=true&cauthor_uid=24911630), [Saldiva P](http://www.ncbi.nlm.nih.gov/pubmed?term=Saldiva P%5BAuthor%5D&cauthor=true&cauthor_uid=24911630), [Yorifuji T](http://www.ncbi.nlm.nih.gov/pubmed?term=Yorifuji T%5BAuthor%5D&cauthor=true&cauthor_uid=24911630), [Loomis D](http://www.ncbi.nlm.nih.gov/pubmed?term=Loomis D%5BAuthor%5D&cauthor=true&cauthor_uid=24911630). Outdoor particulate matter exposure and lung cancer: a systematic review and meta-analysis. [*Environ Health Perspect*.](http://www.ncbi.nlm.nih.gov/pubmed/24911630) 2014;122:906-11.

Harriss DJ, Atkinson G, George K, Cable NT, Reilly T, Haboubi N, Zwahlen M, Egger M, Renehan AG; C-CLEAR group. [Lifestyle factors and colorectal cancer risk (1): systematic review and meta-analysis of associations with body mass index.](http://www.ncbi.nlm.nih.gov/pubmed/19207714) *Colorectal Dis.* 2009;11:547-63.

Hemelt M, Yamamoto H, Cheng KK, Zeegers MP. [The effect of smoking on the male excess of bladder cancer: a meta-analysis and geographical analyses.](http://www.ncbi.nlm.nih.gov/pubmed/18792102) *Int J Cancer. 2009*;124:412-9.

Hjalgrim H, Askling J, Rostgaard K, Hamilton-Dutoit S, Frisch M, Zhang J, Madsen M, Rosdahl N, Konradsen HB, Storm HH, Melbye M. Characteristics of Hodgkin’s Lymphoma after Infectious Mononucleosis. *N Engl J Med*. 2003.349:1324-32.

Hobbs CG, Sterne JA, Bailey M, Heyderman RS, Birchall MA, Thomas SJ. Human papillomavirus and head and neck cancer: a systematic review and meta-analysis. *Clin Otolaryngol*; 2006;31:259-66.

Howel D, Arblaster L, Swinburne L, Schweiger M, Renvoize E, Hatton P. [Routes of asbestos exposure and the development of mesothelioma in an English region.](http://www.ncbi.nlm.nih.gov/pubmed/9245946) *Occup Environ Med.* 1997;54:403-9.

Hsu WL, Chen JY, Chien YC, Liu MY, You SL, Hsu MM, Yang CS, Chen CJ. Independent effect of EBV and cigarette smoking on nasopharyngeal carcinoma: a 20-year follow-up study on 9,622 males without family history in Taiwan. Cancer *Epidemiol Biomarkers Prev.* 2009;18:1218-26.

IOM. Institute of Medicine of the National Academies. Asbestos – Selected Cancers. Committee on Asbestos: selected health effects, board on population health and public health practices. The Washington Academy Press, Washington D.C., 2006.

INH. Inquérito Nacional de Hepatites. Universidade de Pernambuco. Núcleo de Pós-Graduação. *Estudo de prevalência de base populacional das infecções pelos vírus das hepatites A, B e C nas capitais do Brasil*. Relatório de Pesquisa. Brasil, 2010.

<http://www.aids.gov.br/sites/default/files/anexos/publicacao/2010/50071/estudo_prevalencia_hepatites_pdf_26830.pdf>

[Islami F](http://www.ncbi.nlm.nih.gov/pubmed?term=Islami F%5BAuthor%5D&cauthor=true&cauthor_uid=20833578), [Tramacere I](http://www.ncbi.nlm.nih.gov/pubmed?term=Tramacere I%5BAuthor%5D&cauthor=true&cauthor_uid=20833578), [Rota M](http://www.ncbi.nlm.nih.gov/pubmed?term=Rota M%5BAuthor%5D&cauthor=true&cauthor_uid=20833578), [Bagnardi V](http://www.ncbi.nlm.nih.gov/pubmed?term=Bagnardi V%5BAuthor%5D&cauthor=true&cauthor_uid=20833578), [Fedirko V](http://www.ncbi.nlm.nih.gov/pubmed?term=Fedirko V%5BAuthor%5D&cauthor=true&cauthor_uid=20833578), [Scotti L](http://www.ncbi.nlm.nih.gov/pubmed?term=Scotti L%5BAuthor%5D&cauthor=true&cauthor_uid=20833578), [Garavello W](http://www.ncbi.nlm.nih.gov/pubmed?term=Garavello W%5BAuthor%5D&cauthor=true&cauthor_uid=20833578), [Jenab M](http://www.ncbi.nlm.nih.gov/pubmed?term=Jenab M%5BAuthor%5D&cauthor=true&cauthor_uid=20833578), [Corrao G](http://www.ncbi.nlm.nih.gov/pubmed?term=Corrao G%5BAuthor%5D&cauthor=true&cauthor_uid=20833578), [Straif K](http://www.ncbi.nlm.nih.gov/pubmed?term=Straif K%5BAuthor%5D&cauthor=true&cauthor_uid=20833578), [Negri E](http://www.ncbi.nlm.nih.gov/pubmed?term=Negri E%5BAuthor%5D&cauthor=true&cauthor_uid=20833578), [Boffetta P](http://www.ncbi.nlm.nih.gov/pubmed?term=Boffetta P%5BAuthor%5D&cauthor=true&cauthor_uid=20833578), [La Vecchia C](http://www.ncbi.nlm.nih.gov/pubmed?term=La Vecchia C%5BAuthor%5D&cauthor=true&cauthor_uid=20833578). Alcohol drinking and laryngeal cancer: overall and dose-risk relation--a systematic review and meta-analysis. [*Oral Oncol.*](http://www.ncbi.nlm.nih.gov/pubmed/20833578) 2010;46:802-10.

[Islami F](http://www.ncbi.nlm.nih.gov/pubmed?term=Islami F%5BAuthor%5D&cauthor=true&cauthor_uid=21190191), [Fedirko V](http://www.ncbi.nlm.nih.gov/pubmed?term=Fedirko V%5BAuthor%5D&cauthor=true&cauthor_uid=21190191), [Tramacere I](http://www.ncbi.nlm.nih.gov/pubmed?term=Tramacere I%5BAuthor%5D&cauthor=true&cauthor_uid=21190191), [Bagnardi V](http://www.ncbi.nlm.nih.gov/pubmed?term=Bagnardi V%5BAuthor%5D&cauthor=true&cauthor_uid=21190191), [Jenab M](http://www.ncbi.nlm.nih.gov/pubmed?term=Jenab M%5BAuthor%5D&cauthor=true&cauthor_uid=21190191), [Scotti L](http://www.ncbi.nlm.nih.gov/pubmed?term=Scotti L%5BAuthor%5D&cauthor=true&cauthor_uid=21190191), [Rota M](http://www.ncbi.nlm.nih.gov/pubmed?term=Rota M%5BAuthor%5D&cauthor=true&cauthor_uid=21190191), [Corrao G](http://www.ncbi.nlm.nih.gov/pubmed?term=Corrao G%5BAuthor%5D&cauthor=true&cauthor_uid=21190191), [Garavello W](http://www.ncbi.nlm.nih.gov/pubmed?term=Garavello W%5BAuthor%5D&cauthor=true&cauthor_uid=21190191), [Schüz J](http://www.ncbi.nlm.nih.gov/pubmed?term=Schüz J%5BAuthor%5D&cauthor=true&cauthor_uid=21190191), [Straif K](http://www.ncbi.nlm.nih.gov/pubmed?term=Straif K%5BAuthor%5D&cauthor=true&cauthor_uid=21190191), [Negri E](http://www.ncbi.nlm.nih.gov/pubmed?term=Negri E%5BAuthor%5D&cauthor=true&cauthor_uid=21190191), [Boffetta P](http://www.ncbi.nlm.nih.gov/pubmed?term=Boffetta P%5BAuthor%5D&cauthor=true&cauthor_uid=21190191), [La Vecchia C](http://www.ncbi.nlm.nih.gov/pubmed?term=La Vecchia C%5BAuthor%5D&cauthor=true&cauthor_uid=21190191). Alcohol drinking and esophageal squamous cell carcinoma with focus on light-drinkers and never-smokers: a systematic review and meta-analysis. [*Int J Cancer*.](http://www.ncbi.nlm.nih.gov/pubmed/21190191) 2011;129:2473-84.

[Khalade](https://www.ncbi.nlm.nih.gov/pubmed?term=Khalade A%5BAuthor%5D&cauthor=true&cauthor_uid=20584305)A, [Jaakkola MS](https://www.ncbi.nlm.nih.gov/pubmed?term=Jaakkola MS%5BAuthor%5D&cauthor=true&cauthor_uid=20584305), [Pukkala E](https://www.ncbi.nlm.nih.gov/pubmed?term=Pukkala E%5BAuthor%5D&cauthor=true&cauthor_uid=20584305), [Jaakkola JJ](https://www.ncbi.nlm.nih.gov/pubmed?term=Jaakkola JJ%5BAuthor%5D&cauthor=true&cauthor_uid=20584305). Exposure to benzene at work and the risk of leukemia: a systematic review and meta-analysis. [*Environ Health*.](https://www.ncbi.nlm.nih.gov/pubmed/?term=Khalade+AND+Pukkala+AND+benzene+AND+meta-analysis) 2010;9:31.

[Kim CH](http://www.ncbi.nlm.nih.gov/pubmed/?term=Kim CH%5BAuthor%5D&cauthor=true&cauthor_uid=24615328), [Lee YC](http://www.ncbi.nlm.nih.gov/pubmed/?term=Lee YC%5BAuthor%5D&cauthor=true&cauthor_uid=24615328), [Hung RJ](http://www.ncbi.nlm.nih.gov/pubmed/?term=Hung RJ%5BAuthor%5D&cauthor=true&cauthor_uid=24615328), [McNallan SR](http://www.ncbi.nlm.nih.gov/pubmed/?term=McNallan SR%5BAuthor%5D&cauthor=true&cauthor_uid=24615328), [Cote ML](http://www.ncbi.nlm.nih.gov/pubmed/?term=Cote ML%5BAuthor%5D&cauthor=true&cauthor_uid=24615328), [Lim WY](http://www.ncbi.nlm.nih.gov/pubmed/?term=Lim WY%5BAuthor%5D&cauthor=true&cauthor_uid=24615328), [Chang SC](http://www.ncbi.nlm.nih.gov/pubmed/?term=Chang SC%5BAuthor%5D&cauthor=true&cauthor_uid=24615328), [Kim JH](http://www.ncbi.nlm.nih.gov/pubmed/?term=Kim JH%5BAuthor%5D&cauthor=true&cauthor_uid=24615328), [Ugolini D](http://www.ncbi.nlm.nih.gov/pubmed/?term=Ugolini D%5BAuthor%5D&cauthor=true&cauthor_uid=24615328), [Chen Y](http://www.ncbi.nlm.nih.gov/pubmed/?term=Chen Y%5BAuthor%5D&cauthor=true&cauthor_uid=24615328), [Liloglou T](http://www.ncbi.nlm.nih.gov/pubmed/?term=Liloglou T%5BAuthor%5D&cauthor=true&cauthor_uid=24615328), [Andrew AS](http://www.ncbi.nlm.nih.gov/pubmed/?term=Andrew AS%5BAuthor%5D&cauthor=true&cauthor_uid=24615328) et al.. . Exposure to secondhand tobacco smoke and lung cancer by histological type: a pooled analysis of the International Lung Cancer Consortium (ILCCO). [*Int J Cancer.*](http://www.ncbi.nlm.nih.gov/pubmed/?term=Kim+ANd+Lee+AND+Hung+AND+McNallan+AND+cancer) 2014;135:1918-1930.

Kogevinas M, Sala M, Boffetta P, Kazerouni N, Kromhout H, Hoar-Zahm S. [Cancer risk in the rubber industry: a review of the recent epidemiological evidence.](http://www.ncbi.nlm.nih.gov/pubmed/9536156) *Occup Environ Med.* 1998;55:1-12.

Kurihara N, Wada O. [Silicosis and smoking strongly increase lung cancer risk in silica-exposed workers.](http://www.ncbi.nlm.nih.gov/pubmed/15295901) *Ind Health.* 2004;42:303-14.

Larsson SC, Orsini N, Wolk A., Processed meat consumption and stomach cancer risk: a meta-analysis. *J Natl Cancer Inst.* 2006;98:1078-87.

Larsson SC, Wolk A., Obesity and the risk of gallbladder cancer: a meta-analysis. *Br J Cancer*. 2007;96:1457-61.

[Lee YC](http://www.ncbi.nlm.nih.gov/pubmed?term=Lee YC%5BAuthor%5D&cauthor=true&cauthor_uid=19720726), [Cohet C](http://www.ncbi.nlm.nih.gov/pubmed?term=Cohet C%5BAuthor%5D&cauthor=true&cauthor_uid=19720726), [Yang YC](http://www.ncbi.nlm.nih.gov/pubmed?term=Yang YC%5BAuthor%5D&cauthor=true&cauthor_uid=19720726), [Stayner L](http://www.ncbi.nlm.nih.gov/pubmed?term=Stayner L%5BAuthor%5D&cauthor=true&cauthor_uid=19720726), [Hashibe M](http://www.ncbi.nlm.nih.gov/pubmed?term=Hashibe M%5BAuthor%5D&cauthor=true&cauthor_uid=19720726), [Straif K](http://www.ncbi.nlm.nih.gov/pubmed?term=Straif K%5BAuthor%5D&cauthor=true&cauthor_uid=19720726). Meta-analysis of epidemiologic studies on cigarette smoking and liver cancer. [*Int J Epidemiol*.](http://www.ncbi.nlm.nih.gov/pubmed/19720726) 2009;38:1497-511.

Lenters V, Vermeulen R, Dogger S, Stayner L, Portengen L, Burdof A, Heederik D.   A Meta-analysis of Asbestos and Lung Cancer: Is Better Quality Exposure Assessment Associated with Steeper Slopes of the Exposure–Response Relationships?  *Environmental Health Perspectives*. 2011;119:1547-55.

[Lipsett M](http://www.ncbi.nlm.nih.gov/pubmed/?term=Lipsett M%5BAuthor%5D&cauthor=true&cauthor_uid=10394308), [Campleman S](http://www.ncbi.nlm.nih.gov/pubmed/?term=Campleman S%5BAuthor%5D&cauthor=true&cauthor_uid=10394308). Occupational exposure to diesel exhaust and lung cancer: a meta-analysis. [*Am J Public Health*.1999;89:1009-17.](http://www.ncbi.nlm.nih.gov/pubmed/?term=Lipsett+AND+Campleman+AND+1999+AND+cancer)

[Liu Y](http://www.ncbi.nlm.nih.gov/pubmed/?term=Liu Y%5BAuthor%5D&cauthor=true&cauthor_uid=21802197), [Hu F](http://www.ncbi.nlm.nih.gov/pubmed/?term=Hu F%5BAuthor%5D&cauthor=true&cauthor_uid=21802197), [Li D](http://www.ncbi.nlm.nih.gov/pubmed/?term=Li D%5BAuthor%5D&cauthor=true&cauthor_uid=21802197), [Wang F](http://www.ncbi.nlm.nih.gov/pubmed/?term=Wang F%5BAuthor%5D&cauthor=true&cauthor_uid=21802197), [Zhu L](http://www.ncbi.nlm.nih.gov/pubmed/?term=Zhu L%5BAuthor%5D&cauthor=true&cauthor_uid=21802197), [Chen W](http://www.ncbi.nlm.nih.gov/pubmed/?term=Chen W%5BAuthor%5D&cauthor=true&cauthor_uid=21802197), [Ge J](http://www.ncbi.nlm.nih.gov/pubmed/?term=Ge J%5BAuthor%5D&cauthor=true&cauthor_uid=21802197), [An R](http://www.ncbi.nlm.nih.gov/pubmed/?term=An R%5BAuthor%5D&cauthor=true&cauthor_uid=21802197), [Zhao Y](http://www.ncbi.nlm.nih.gov/pubmed/?term=Zhao Y%5BAuthor%5D&cauthor=true&cauthor_uid=21802197). Does physical activity reduce the risk of prostate cancer? A systematic review and meta-analysis. [*Eur Urol.*](http://www.ncbi.nlm.nih.gov/pubmed/?term=YuPeng+Liu+et+al.+Eur+Urol%2C+2011.) 2011;60:1029-44.

Moore SC, Gierach GL, Schatzkin A, Matthews CE. Physical activity, sedentary behaviours, and the prevention of endometrial cancer. *Br J Cancer.* 2010;103:933-8.

[O'Rorke MA](http://www.ncbi.nlm.nih.gov/pubmed/?term=O'Rorke MA%5BAuthor%5D&cauthor=true&cauthor_uid=19856317), [Cantwell MM](http://www.ncbi.nlm.nih.gov/pubmed/?term=Cantwell MM%5BAuthor%5D&cauthor=true&cauthor_uid=19856317), [Cardwell CR](http://www.ncbi.nlm.nih.gov/pubmed/?term=Cardwell CR%5BAuthor%5D&cauthor=true&cauthor_uid=19856317), [Mulholland HG](http://www.ncbi.nlm.nih.gov/pubmed/?term=Mulholland HG%5BAuthor%5D&cauthor=true&cauthor_uid=19856317), [Murray LJ](http://www.ncbi.nlm.nih.gov/pubmed/?term=Murray LJ%5BAuthor%5D&cauthor=true&cauthor_uid=19856317). Can physical activity modulate pancreatic cancer risk? a systematic review and meta-analysis*.* [*Int J Cancer.*](http://www.ncbi.nlm.nih.gov/pubmed/?term=O’Rorke+et+al%2C+Int+J+Cancer%2C+2010.) 2010;126:2957-68.

[Pavia M](http://www.ncbi.nlm.nih.gov/pubmed/?term=Pavia M%5BAuthor%5D&cauthor=true&cauthor_uid=16685056), [Pileggi C](http://www.ncbi.nlm.nih.gov/pubmed/?term=Pileggi C%5BAuthor%5D&cauthor=true&cauthor_uid=16685056), [Nobile CG](http://www.ncbi.nlm.nih.gov/pubmed/?term=Nobile CG%5BAuthor%5D&cauthor=true&cauthor_uid=16685056), [Angelillo IF](http://www.ncbi.nlm.nih.gov/pubmed/?term=Angelillo IF%5BAuthor%5D&cauthor=true&cauthor_uid=16685056). Association between fruit and vegetable consumption and oral cancer: a meta-analysis of observational studies. [*Am J Clin Nutr.*](http://www.ncbi.nlm.nih.gov/pubmed/?term=Pavia+et+al.+Am+J+Clin+Nutr.+2006+May%3B83(5)%3A1126-34.) 2006;83:1126-34.

[Pesch B](http://www.ncbi.nlm.nih.gov/pubmed/?term=Pesch B%5BAuthor%5D&cauthor=true&cauthor_uid=17881467), [Pierl CB](http://www.ncbi.nlm.nih.gov/pubmed/?term=Pierl CB%5BAuthor%5D&cauthor=true&cauthor_uid=17881467), [Gebel M](http://www.ncbi.nlm.nih.gov/pubmed/?term=Gebel M%5BAuthor%5D&cauthor=true&cauthor_uid=17881467), [Gross I](http://www.ncbi.nlm.nih.gov/pubmed/?term=Gross I%5BAuthor%5D&cauthor=true&cauthor_uid=17881467), [Becker D](http://www.ncbi.nlm.nih.gov/pubmed/?term=Becker D%5BAuthor%5D&cauthor=true&cauthor_uid=17881467), [Johnen G](http://www.ncbi.nlm.nih.gov/pubmed/?term=Johnen G%5BAuthor%5D&cauthor=true&cauthor_uid=17881467), [Rihs HP](http://www.ncbi.nlm.nih.gov/pubmed/?term=Rihs HP%5BAuthor%5D&cauthor=true&cauthor_uid=17881467), [Donhuijsen K](http://www.ncbi.nlm.nih.gov/pubmed/?term=Donhuijsen K%5BAuthor%5D&cauthor=true&cauthor_uid=17881467), [Lepentsiotis V](http://www.ncbi.nlm.nih.gov/pubmed/?term=Lepentsiotis V%5BAuthor%5D&cauthor=true&cauthor_uid=17881467), [Meier M](http://www.ncbi.nlm.nih.gov/pubmed/?term=Meier M%5BAuthor%5D&cauthor=true&cauthor_uid=17881467), [Schulze J](http://www.ncbi.nlm.nih.gov/pubmed/?term=Schulze J%5BAuthor%5D&cauthor=true&cauthor_uid=17881467), [Brüning T](http://www.ncbi.nlm.nih.gov/pubmed/?term=Brüning T%5BAuthor%5D&cauthor=true&cauthor_uid=17881467).

Occupational risks for adenocarcinoma of the nasal cavity and paranasal sinuses in the German wood industry. [*Occup Environ Med*.](http://www.ncbi.nlm.nih.gov/pubmed/17881467) 2008;65:191-6.

PETAb, 2008. *Pesquisa Especial sobre Tabagismo* (PETab 2008). Instituto Brasileiro de Geografia e Estatística (IBGE). Rio de Janeiro: IBGE, 2009.

<http://www.ibge.gov.br/home/estatistica/populacao/trabalhoerendimento/pnad2008/suplementos/tabagismo/>

PNAD, 2003*. Pesquisa Nacional por Amostra de Domicílios (PNAD 2003).* Instituto Brasileiro de Geografia e Estatística (IBGE). Rio de Janeiro: IBGE, 2004.

<http://www.ibge.gov.br/home/estatistica/populacao/trabalhoerendimento/pnad2003/coeficiente_brasil.shtm>

PNAD, 2008*. Pesquisa Nacional por Amostra de Domicílios (PNAD 2008).* Instituto Brasileiro de Geografia e Estatística (IBGE). Rio de Janeiro: IBGE, 2010.

<http://www.ibge.gov.br/home/estatistica/populacao/trabalhoerendimento/pnad2008/microdados.shtm>

PNDS, 2009. *Pesquisa Nacional Demografia e Saúde da Criança e da Mulher* (PNDS-2006). Ministério da Saúde. Brasília: Ministério da Saúde, 2009.

<http://bvsms.saude.gov.br/bvs/pnds/index.php>

POF. *Pesquisa de Orçamentos Familiares 2008-2009: despesas, rendimentos e condições de vida.*Instituto Brasileiro de Geografia e Estatística (IBGE). Rio de Janeiro: IBGE, 2010.

<http://www.ibge.gov.br/home/estatistica/populacao/condicaodevida/pof/2008_2009_analise_consumo/default.shtm>

[Reeves GK](http://www.ncbi.nlm.nih.gov/pubmed?term=Reeves GK%5BAuthor%5D&cauthor=true&cauthor_uid=19190634), [Pirie K](http://www.ncbi.nlm.nih.gov/pubmed?term=Pirie K%5BAuthor%5D&cauthor=true&cauthor_uid=19190634), [Green J](http://www.ncbi.nlm.nih.gov/pubmed?term=Green J%5BAuthor%5D&cauthor=true&cauthor_uid=19190634), [Bull D](http://www.ncbi.nlm.nih.gov/pubmed?term=Bull D%5BAuthor%5D&cauthor=true&cauthor_uid=19190634), [Beral V](http://www.ncbi.nlm.nih.gov/pubmed?term=Beral V%5BAuthor%5D&cauthor=true&cauthor_uid=19190634); [Million Women Study Collaborators](http://www.ncbi.nlm.nih.gov/pubmed?term=Million Women Study Collaborators%5BCorporate Author%5D). Reproductive factors and specific histological types of breast cancer: prospective study and meta-analysis. [*Br J Cancer.*](http://www.ncbi.nlm.nih.gov/pubmed/19190634)2009;100:538-44.

Renehan AG, Tyson M, Egger M, Heller RF, Zwahlen M. Body-mass index and incidence of cancer: a systematic review and meta-analysis of prospective observational studies*. Lancet.* 2008;371:569-78.

Riboli E, Norat T. Epidemiologic evidence of the protective effect of fruit and vegetables on cancer risk. *Am J Clin Nutr.* 2003;78:559S-569S.

Shikata K, Kiyohara Y, Kubo M, Yonemoto K, Ninomiya T, Shirota T, Tanizaki Y, Doi Y, Tanaka K, Oishi Y, Matsumoto T, Iida M., A prospective study of dietary salt intake and gastric cancer incidence in a defined Japanese population: The Hisayama study. *Int. J. Cancer.* 2006;119:196–201.

Thun MJ, Apicella LF, Henley SJ. Smoking vs other risk factors as the cause of smoking-attributable mortality: confounding in the courtroom. *JAMA.* 2000;284:706–12.

[Tramacere I](http://www.ncbi.nlm.nih.gov/pubmed?term=Tramacere I%5BAuthor%5D&cauthor=true&cauthor_uid=21536659), [Negri E](http://www.ncbi.nlm.nih.gov/pubmed?term=Negri E%5BAuthor%5D&cauthor=true&cauthor_uid=21536659), [Pelucchi C](http://www.ncbi.nlm.nih.gov/pubmed?term=Pelucchi C%5BAuthor%5D&cauthor=true&cauthor_uid=21536659), [Bagnardi V](http://www.ncbi.nlm.nih.gov/pubmed?term=Bagnardi V%5BAuthor%5D&cauthor=true&cauthor_uid=21536659), [Rota M](http://www.ncbi.nlm.nih.gov/pubmed?term=Rota M%5BAuthor%5D&cauthor=true&cauthor_uid=21536659), [Scotti L](http://www.ncbi.nlm.nih.gov/pubmed?term=Scotti L%5BAuthor%5D&cauthor=true&cauthor_uid=21536659), [Islami F](http://www.ncbi.nlm.nih.gov/pubmed?term=Islami F%5BAuthor%5D&cauthor=true&cauthor_uid=21536659), [Corrao G](http://www.ncbi.nlm.nih.gov/pubmed?term=Corrao G%5BAuthor%5D&cauthor=true&cauthor_uid=21536659), [La Vecchia C](http://www.ncbi.nlm.nih.gov/pubmed?term=La Vecchia C%5BAuthor%5D&cauthor=true&cauthor_uid=21536659), [Boffetta P](http://www.ncbi.nlm.nih.gov/pubmed?term=Boffetta P%5BAuthor%5D&cauthor=true&cauthor_uid=21536659). A meta-analysis on alcohol drinking and gastric cancer risk. [*Ann Oncol.*](http://www.ncbi.nlm.nih.gov/pubmed/21536659)2012;23:28-36.

[Turati F](http://www.ncbi.nlm.nih.gov/pubmed?term=Turati F%5BAuthor%5D&cauthor=true&cauthor_uid=22949102), [Garavello W](http://www.ncbi.nlm.nih.gov/pubmed?term=Garavello W%5BAuthor%5D&cauthor=true&cauthor_uid=22949102), [Tramacere I](http://www.ncbi.nlm.nih.gov/pubmed?term=Tramacere I%5BAuthor%5D&cauthor=true&cauthor_uid=22949102), [Pelucchi C](http://www.ncbi.nlm.nih.gov/pubmed?term=Pelucchi C%5BAuthor%5D&cauthor=true&cauthor_uid=22949102), [Galeone C](http://www.ncbi.nlm.nih.gov/pubmed?term=Galeone C%5BAuthor%5D&cauthor=true&cauthor_uid=22949102), [Bagnardi V](http://www.ncbi.nlm.nih.gov/pubmed?term=Bagnardi V%5BAuthor%5D&cauthor=true&cauthor_uid=22949102), [Corrao G](http://www.ncbi.nlm.nih.gov/pubmed?term=Corrao G%5BAuthor%5D&cauthor=true&cauthor_uid=22949102), [Islami F](http://www.ncbi.nlm.nih.gov/pubmed?term=Islami F%5BAuthor%5D&cauthor=true&cauthor_uid=22949102), [Fedirko V](http://www.ncbi.nlm.nih.gov/pubmed?term=Fedirko V%5BAuthor%5D&cauthor=true&cauthor_uid=22949102), [Boffetta P](http://www.ncbi.nlm.nih.gov/pubmed?term=Boffetta P%5BAuthor%5D&cauthor=true&cauthor_uid=22949102), [La Vecchia C](http://www.ncbi.nlm.nih.gov/pubmed?term=La Vecchia C%5BAuthor%5D&cauthor=true&cauthor_uid=22949102), [Negri E](http://www.ncbi.nlm.nih.gov/pubmed?term=Negri E%5BAuthor%5D&cauthor=true&cauthor_uid=22949102). A meta-analysis of alcohol drinking and oral and pharyngeal cancers: results from subgroup analyses. [*Alcohol Alcohol.*](http://www.ncbi.nlm.nih.gov/pubmed/22949102) 2013;48:107-18.

[Ursin G](http://www.ncbi.nlm.nih.gov/pubmed?term=Ursin G%5BAuthor%5D&cauthor=true&cauthor_uid=12439712), [Yu MC](http://www.ncbi.nlm.nih.gov/pubmed?term=Yu MC%5BAuthor%5D&cauthor=true&cauthor_uid=12439712), [Longnecker MP](http://www.ncbi.nlm.nih.gov/pubmed?term=Longnecker MP%5BAuthor%5D&cauthor=true&cauthor_uid=12439712), [Newcomb P](http://www.ncbi.nlm.nih.gov/pubmed?term=Newcomb P%5BAuthor%5D&cauthor=true&cauthor_uid=12439712), [Bergkvist L](http://www.ncbi.nlm.nih.gov/pubmed?term=Bergkvist L%5BAuthor%5D&cauthor=true&cauthor_uid=12439712), [Kalache A](http://www.ncbi.nlm.nih.gov/pubmed?term=Kalache A%5BAuthor%5D&cauthor=true&cauthor_uid=12439712), [Farley TM](http://www.ncbi.nlm.nih.gov/pubmed?term=Farley TM%5BAuthor%5D&cauthor=true&cauthor_uid=12439712), [Holck S](http://www.ncbi.nlm.nih.gov/pubmed?term=Holck S%5BAuthor%5D&cauthor=true&cauthor_uid=12439712), [Meirik O](http://www.ncbi.nlm.nih.gov/pubmed?term=Meirik O%5BAuthor%5D&cauthor=true&cauthor_uid=12439712); [Collaborative Group on Hormonal Factors in Breast Cancer](http://www.ncbi.nlm.nih.gov/pubmed?term=Collaborative Group on Hormonal Factors in Breast Cancer%5BCorporate Author%5D). Alcohol, tobacco and breast cancer--collaborative reanalysis of individual data from 53 epidemiological studies, including 58,515 women with breast cancer and 95,067 women without the disease. [*Br J Cancer.*](http://www.ncbi.nlm.nih.gov/pubmed/12439712) 2002 Nov 18;87:1234-45.

VIGITEL. V*igilância de Fatores de Risco e Proteção para Doenças Crônicas por Inquérito Telefônico-Brasil, 2008.* Ministério da Saúde. Brasília: Ministério da Saúde, 2009.

<http://bvsms.saude.gov.br/bvs/publicacoes/vigitel_brasil_2008.pdf>

Xue FB, Xu YY, Wan Y,Pan BR,Ren J,Fan DM. Association of H pylori infection with gastric carcinoma: a meta-analysis. *World J Gastroenterol.* 2001;7:801-4.

[Wang JX](http://www.ncbi.nlm.nih.gov/pubmed/?term=Wang JX%5BAuthor%5D&cauthor=true&cauthor_uid=11906134), [Zhang LA](http://www.ncbi.nlm.nih.gov/pubmed/?term=Zhang LA%5BAuthor%5D&cauthor=true&cauthor_uid=11906134), [Li BX](http://www.ncbi.nlm.nih.gov/pubmed/?term=Li BX%5BAuthor%5D&cauthor=true&cauthor_uid=11906134), [Zhao YC](http://www.ncbi.nlm.nih.gov/pubmed/?term=Zhao YC%5BAuthor%5D&cauthor=true&cauthor_uid=11906134), [Wang ZQ](http://www.ncbi.nlm.nih.gov/pubmed/?term=Wang ZQ%5BAuthor%5D&cauthor=true&cauthor_uid=11906134), [Zhang JY](http://www.ncbi.nlm.nih.gov/pubmed/?term=Zhang JY%5BAuthor%5D&cauthor=true&cauthor_uid=11906134), [Aoyama T](http://www.ncbi.nlm.nih.gov/pubmed/?term=Aoyama T%5BAuthor%5D&cauthor=true&cauthor_uid=11906134). Cancer incidence and risk estimation among medical x-ray workers in China, 1950-1995. [Health Phys.](http://www.ncbi.nlm.nih.gov/pubmed/11906134) 2002;82:455-6.

Weiland SK, Straif K, Chambless L, Werner B, Mundt KA, Bucher A, Birk T, Keil U.[Workplace risk factors for cancer in the German rubber industry: Part 1. Mortality from respiratory cancers.](http://www.ncbi.nlm.nih.gov/pubmed/9764109) *Occup Environ Med*. 1998;55(5):317-24.

Wolin KY, Yan Y, Colditz GA, Lee IM. Physical activity and colon cancer prevention: a meta-analysis. *Br J Cancer*. 2009;100:611-6.

World Cancer Research Fund / American Institute for Cancer Research., *Food, Nutrition, Physical Activity, and the Prevention of Cancer: a Global Perspective.* Washington DC: AICR, 2007.

Wu Y, Zhang D, Kang S. Physical activity and risk of breast cancer: a meta-analysis of prospective studies. *Breast Cancer Res Treat.* 2013;137:869-82.

Wyss A, Hashibe M, Chuang SC, Lee YC, Zhang ZF, Yu GP, Winn DM, Wei Q, Talamini R, Szeszenia-Dabrowska N, Sturgis EM, Smith E et al. [Cigarette, cigar, and pipe smoking and the risk of head and neck cancers: pooled analysis in the International Head and Neck Cancer Epidemiology Consortium.](http://www.ncbi.nlm.nih.gov/pubmed/23817919) *Am J Epidemiol.* 2013;178:679-90.

[Zhang L](http://www.ncbi.nlm.nih.gov/pubmed/?term=Zhang L%5BAuthor%5D&cauthor=true&cauthor_uid=18674636), [Steinmaus C](http://www.ncbi.nlm.nih.gov/pubmed/?term=Steinmaus C%5BAuthor%5D&cauthor=true&cauthor_uid=18674636), [Eastmond DA](http://www.ncbi.nlm.nih.gov/pubmed/?term=Eastmond DA%5BAuthor%5D&cauthor=true&cauthor_uid=18674636), [Xin XK](http://www.ncbi.nlm.nih.gov/pubmed/?term=Xin XK%5BAuthor%5D&cauthor=true&cauthor_uid=18674636), [Smith MT](http://www.ncbi.nlm.nih.gov/pubmed/?term=Smith MT%5BAuthor%5D&cauthor=true&cauthor_uid=18674636). Formaldehyde exposure and leukemia: a new meta-analysis and potential mechanisms. [*Mutat Res.*](http://www.ncbi.nlm.nih.gov/pubmed/18674636) 2009;681:150-68.
